# Regulation of DNA Replication through Natural Impediments in the Eukaryotic Genome

**DOI:** 10.3390/genes8030098

**Published:** 2017-03-07

**Authors:** Mariana C. Gadaleta, Eishi Noguchi

**Affiliations:** Department of Biochemistry and Molecular Biology, Drexel University College of Medicine, Philadelphia, PA 19102, USA; gadaleta@scripps.edu

**Keywords:** DNA replication, replication fork, difficult-to-replicate, replication machinery, replisome, natural impediments, DNA damage, repetitive DNA, secondary structures, genomic instability

## Abstract

All living organisms need to duplicate their genetic information while protecting it from unwanted mutations, which can lead to genetic disorders and cancer development. Inaccuracies during DNA replication are the major cause of genomic instability, as replication forks are prone to stalling and collapse, resulting in DNA damage. The presence of exogenous DNA damaging agents as well as endogenous difficult-to-replicate DNA regions containing DNA–protein complexes, repetitive DNA, secondary DNA structures, or transcribing RNA polymerases, increases the risk of genomic instability and thus threatens cell survival. Therefore, understanding the cellular mechanisms required to preserve the genetic information during S phase is of paramount importance. In this review, we will discuss our current understanding of how cells cope with these natural impediments in order to prevent DNA damage and genomic instability during DNA replication.

## 1. Introduction

DNA replication is essential in all living organisms. It is highly complex and regulated at many levels to ensure accurate and timely duplication of genetic information. Defects in the pathways involved in DNA synthesis and/or repair can lead to mutagenesis and chromosomal rearrangements, both of which are central causes for cancer, aging, and other genetic diseases.

Aside from the DNA damage events that happen under physiological conditions [1], eukaryotic genomes themselves present a wide range of natural impediments to DNA replication [2]. A subset of these impediments, called replication fork barriers (RFBs), can slow down or stall the progression of the replication machinery. If not properly regulated, RFBs could lead to fork collapse and a consequent increase in the susceptibility to DNA double strand breaks (DSBs). RFBs can arise from inherently difficult-to-replicate DNA sequences that form secondary structures, such as repetitive and palindromic DNA sequences. In addition, RFBs can originate from a variety of complexes formed by DNA and non-nucleosomal proteins present along the eukaryotic genome [3]. In eukaryotes, RFBs can be found at telomeres, centromeres, highly transcribed genes, and origins of replication, among other locations [2]. Furthermore, some of these RFBs act as programmed polar pausing sites for the replication machinery in order to control other biological processes, such as mating-type switching in *Schizosaccharomyces pombe* [4]. Understanding how the replication machinery copes with such a variety of circumstances in each round of DNA replication is a subject of intense research.

Another type of natural impediment that occurs during DNA replication is the encounter between the replication machinery and other enzymatic complexes that also operate on the DNA. Particularly, numerous studies have demonstrated that encounters between the replisome and the RNA polymerase II (RNA Pol II) complex cause replication stress and genomic instability [5]. In addition, the transcription process itself could be a source of DNA damage. This is, in part, due to the inherent process of DNA unwinding and exposure of ssDNA that contributes to mutations and DNA damage followed by recombination events.

Here we provide an overview of the major types of natural impediments found by the replication machinery. We will first discuss RFBs formed by repetitive DNA sequences and non-histone DNA-binding proteins. We will then focus on the mechanisms that regulate the coordination between transcription and replication machineries, as collisions between the two machineries may result in transcription-associated recombination and mutagenesis. Supplementary Table S1 summarizes a variety of replication impediments in humans, *Schizosaccharomyces pombe*, and *Saccharomyces cerevisiae*. 

## 2. Replication Barriers Associated with Repeat DNA and Protein–DNA Complexes

RFBs have been reported at rDNA arrays, centromeres, telomeres, mating-type locus, and tRNA genes [6]. In some cases, RFBs may function to coordinate replication and transcription processes and/or to prevent replication slippage at repetitive DNA loci. Other RFBs are genetically programed to establish a site where genomic instability is created under tight cellular control in order to achieve cellular differentiation.

### 2.1. Telomeres

**Figure 1:**
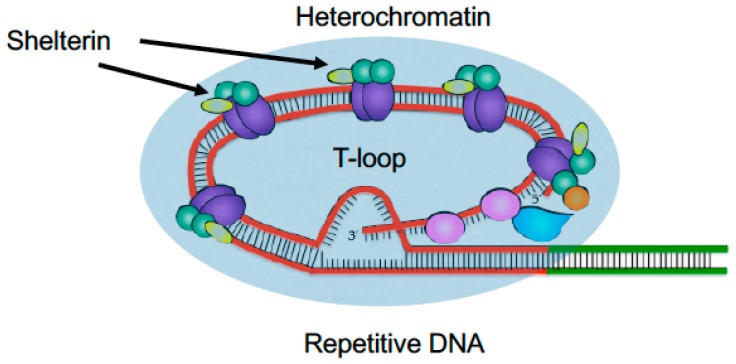
Telomeres are difficult to replicate. The telomere features including heterochromatin, shelterin proteins, and repeat DNA can hamper progression of the replication fork. The red and green sections in the telomere represent telomeric and subtelomeric sequences, respectively. Although in vivo T-loop formation has not been confirmed in yeast, this structure has been described to form in order to protect the 3′ end overhang from recognition by the DNA repair machinery.

Telomeres are the physical ends of eukaryotic chromosomes. They provide a mechanism for full replication of the chromosomal ends while protecting these ends from DNA degradation and recombination. At the same time, several telomeric features pose challenges to the replication machinery (Figure 1). At telomeres, replication forks proceed in a unidirectional manner from a centromere-proximal origin [7,8]. Although some later reports suggest that replication could also be initiated within the telomeric repeats [9,10,11], the unidirectional nature of telomere replication may present a problem for complete replication of chromosomal ends [12]. Furthermore, the repetitive nature of their DNA sequence, the presence of heterochromatin proteins and chromatin remodeling marks, and the potential to form T-loop structures make telomeres difficult-to-replicate templates for DNA replication (Figure 1).

In both *S. pombe* and *S. cerevisiae*, telomeric repeat DNA sequences can slow down replication fork progression at their native loci (chromosomal ends) and also at internal chromosomal regions when tracts of telomeric repeats are artificially inserted [7,13]. Although the nature of the telomeric replication barrier is not well understood, Gadaleta et al. recently showed that the repetitive nature of the telomeric DNA, but not other telomeric features, is the major cause for unstable replisomes in the absence of Swi1 [14]. Swi1 is a subunit of the fork protection complex (FPC: S. *pombe* Swi1–Swi3; *S. cerevisiae* Tof1–Csm3; metazoan Timeless–Tipin). The FPC travels with the replisome in order to protect replication fork structures at various RFBs [15,16]. Interestingly, the same study showed that *swi1*∆ mutants display increased DNA damage and recombination at telomeres, leading to activation of ALT-like pathways of telomere maintenance, suggesting a role of Swi1 as an anti-recombinase at the telomere [14,17]. Consistently, depletion of Timeless (human homolog of Swi1) results in increased levels of DNA repair foci and sister chromatid exchange in mouse cells, indicative of elevated levels of homologous recombination [18]. Furthermore, Timeless interacts with the telomere-binding protein TRF1 and prevents telomere abnormalities in human cells [19], which demonstrates a conserved role of Timeless-related proteins in telomere protection.

In fission yeast, replication through telomeric repeats is facilitated by the telomere-binding protein Taz1, a member of the Myb/SANT DNA-binding domain-containing family of proteins [7,20]. Loss of Taz1 leads to replication fork pausing in the vicinity of telomeres and also at telomere tracts inserted within the chromosome [7]. TRF1, the mammalian homolog of Taz1, is also required for efficient telomere replication [11]. Conflicting data were obtained from experiments done in vitro. In a cell-free SV40 replication system, TRF1 and TRF2 significantly inhibit replication fork progression through the telomeric-repeat tract inserted in a plasmid [19,21]. In addition, overexpression of TRF1 and TRF2 in HeLa cells slowed down replication fork progression through telomeric repeats, suggesting that telomere-binding proteins such as TRF1 and TRF2 obstruct the passage of the replisome [21]. A more recent publication suggests that overexpression of TRF1 in the SV40 system might sequester key replisome factors that are essential for efficient telomere replication, pointing out this observation as the reason for the controversial findings [22].

In *S. cerevisiae*, replication forks stall at telomeric and subtelomeric regions in a manner independent of the repeat orientation [13]. Fork stalling at telomeres likely requires Rap1, while other telomeric and subtelomeric proteins including Reb1, Tbf1, Rif1, Rif2, Sir2, Sir3, and Sir4 are not significant obstacles for replication fork progression [23]. In addition, telomere length can affect the timing of telomere replication: while normal telomeres replicate late in S phase, short telomeres replicate earlier. One of the underlying mechanisms of this timing switch has been recently described. Late replication timing of normal telomeres requires Rif1. However, at short telomeres, Tel1-mediated phosphorylation of Rif1 seems to override this effect and cause early replication of telomeres in budding yeast [24].

G-quadruplexes are another important replication obstacle that can arise at telomeres due to their repetitive GT-rich sequence. Studies have suggested a role for TRF1-related proteins in recruiting DNA helicases to aid in replication of telomeres. These include mammalian RTEL (regulator of telomere length) and BLM (Bloom’s syndrome) helicases, which may resolve G-quadruplex structures during DNA replication [11,25]. In yeast, DNA helicases such as *S. cerevisiae* Pif1 and *S. pombe* Pfh1 suppress G-quadruplex-induced genomic instability and facilitate efficient telomere replication [26,27,28]. Further details on the role of G-quadruplexes as natural replicative obstacles are provided in a later section of this review article.

### 2.2. rDNA Repeats

**Figure 2:**
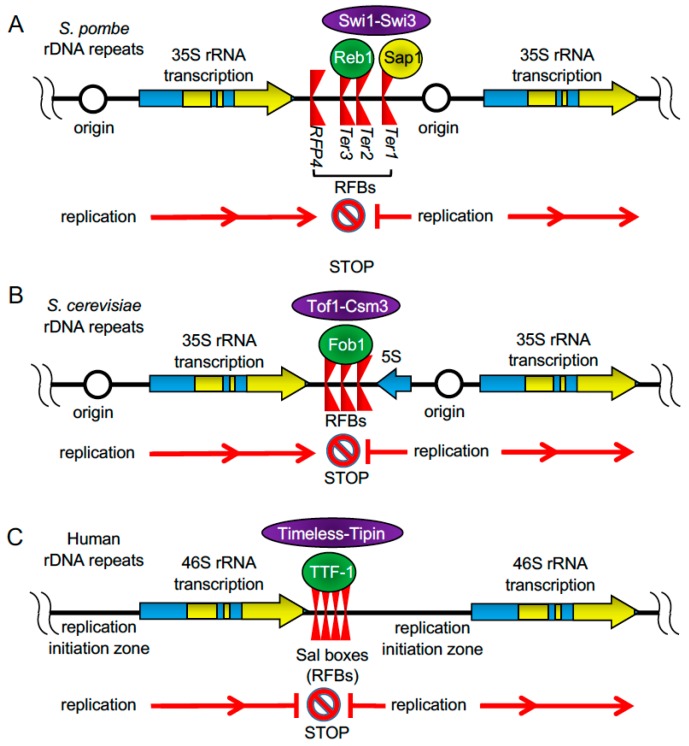
Replication fork barriers (RFBs) at rDNA loci. (**A**) Each fission yeast rDNA repeat contains a replication origin, 35S rRNA transcription unit, and polar fork-block sites (*Ter1*, *Ter2*, *Ter3*, and RFP4). *Ter1*, *Ter2*, and *Ter3* sites block the replication fork in a Swi1–Swi3 dependent manner. Sap1 arrests fork progression at *Ter1*, while Reb1 halts fork progression at *Ter2* and *Ter3*. Fork blockage at RFP4 appears to be dependent on transcription. (**B**) In budding yeast, collisions between the replication fork and transcription machinery are prevented by Fob1-mediated polar fork blockage at RFBs. This fork block requires the function of Tof1-Csm3. (**C**) In mammalian cells, TTF-1, a homolog of fission yeast Reb1, arrests fork progression at multiple Sal boxes located near the 3′ end of the 46S rRNA transcription unit. Unlike the cases in budding and fission yeast, fork progression is blocked from both sides in a manner dependent on Timeless–Tipin.

Ribosomal DNA (rDNA) is found as tandem repeats localized at discreet locations in the genome. Each eukaryotic rDNA transcription unit contains sequences encoding 16–18S rRNA, 5.8S rRNA, and 25–28S rRNA (Figure 2). The transcription units are separated by the non-transcribed spacers, where replication barriers are located in most eukaryotic species, including yeast, ciliates (*Tetrahymena thermophila*), pea (*Pisum sativum*), frog (*Xenopus laevis*), mouse (*Mus musculus*), and humans. During S phase, these barriers lead to replication fork arrest, which is likely to coordinate transcription, replication, and recombination at these loci [29,30,31,32,33,34,35,36,37,38].

A significant volume of research focused on understanding genome stability at rDNA repeats has been carried out using *S. pombe*. The *S. pombe* genome contains 100–150 copies of rDNA repeats at the ends of chromosome III. Each 10.9-kb repeat unit is composed of the 35S rDNA transcriptional unit and a non-transcribed region that contains an origin of replication (*ori3001*) and four closely spaced polar replication barriers (*Ter1/RFB1*, *Ter2/RFB2*, *Ter3/RFB3* and *RFP4*) that block replication forks moving in opposite direction to the transcription machinery (Figure 2A) [38,39,40]. A group of factors is involved in fork pausing at *Ter1–3*. In contrast to *Ter1–3*, no *trans*-acting factor has been identified for *RFP4*, and this barrier is not functional when placed in a plasmid [41]. Two-dimensional gel electrophoresis analysis demonstrated that, in the absence of Reb1, Swi1, or Swi3, the intensity of fork-pausing signal increases at *RFP4*. This suggests that pausing at *RFP4* may be a consequence of replication and transcription machineries colliding at *RFP4* when the three main barriers (*Ter1–3*) fail to pause the replication fork [39].

Replication pausing at *Ter1–3* requires the FPC subunits, Swi1 and Swi3 in *S. pombe* [39,40]. Switch-activating protein 1 (Sap1) and Reb1 function as *trans*-acting elements that cause fork pausing by binding to the replication barriers in the rDNA repeats. Sap1 is responsible for the barrier activity at *Ter1* and is also involved in chromatin formation, checkpoint activation, and genome stability (Figure 2A). Loss of Sap1 causes defects in chromosome segregation, and mutations that affect Sap1-DNA binding in vitro compromise the barrier activity at *Ter1* [41,42,43,44,45,46]. In addition to its role in fork pausing at *Ter1*, Sap1 has barrier activity at long terminal repeats (LTRs) [47]. Sap1 also binds to the *SAS1* region at the mating-type locus, although it does not promote barrier activity at this site, possibly due to lower affinity binding that fails to produce fork stalling at this particular site [41,48].

Reb1 is a member of the Myb/SANT family of proteins and is related to the mammalian transcription termination factor-1 (TTF-1) [39,40]. In *S. pombe*, Reb1 binds to *Ter2–3* in the rDNA (Figure 2A) and *Ter*-like sites present outside the rDNA throughout the genome. Like Sap1, Reb1 acts as a dimer and causes DNA bending when bound to two separate sites in *cis* [49]. When bound to *Ter2–3* at the rDNA repeats, Reb1 not only mediates polar barrier activity for replication forks moving towards the transcription machinery, but also arrests transcription catalyzed by RNA Pol I from the opposite direction [50,51]. This is different from Sap1, which may not affect the progression of the transcription machinery. Later work showed that Reb1 binds to *Ter* sites located outside the rDNA, where it promotes DNA looping between two *Ter* sites. This type of inter-chromosomal interactions, called “chromosome kissing” appears to cause transcription and replication termination [49,52]. Interestingly, Reb1 also functions as an activator of RNA Pol II-dependent transcription at certain promoters [49,53].

Budding yeast Fob1 regulates rDNA recombination by causing polar replication fork arrest at RFB sites (Figure 2B), by facilitating protein-mediated chromosome kissing [54]. Fob1 also recruits silencing factors such as Sir2 and the RENT complex to the same sites [55,56,57,58,59]. However, Fob1’s function in rDNA silencing appears to be independent on its role in fork arrest; when the *S. cerevisiae* FPC components Tof1 or Csm3 are inactivated, fork pausing is lost at the RFB sites, but the silencing activity of Fob1 remains intact [60]. Interestingly, *S. pombe* Reb1 tethers the mating-type locus to rDNA *Ter* sites in order to facilitate gene silencing of the mating-type locus through heterochromatin formation [61]. Therefore, although Reb1 and Fob1 fail to show structural similarities at the level of amino acid sequences, these two proteins share common functions, which are promoted by chromosome kissing. Further investigation will shed light on how Reb1 and Fob1, through chromosome kissing, aid in the coordination of transcription and replication processes.

In mice and human cells, replication fork arrest occurs within repeated regions called Sal boxes located downstream from the ribosomal 47S pre-rRNA-coding region (Figure 2C) [62,63]. Sal boxes recruit TTF-1, the mammalian ortholog of *S. pombe* Reb1, which is involved in termination of pre-mRNA transcription [64]. As is the case for *S. pombe* Swi1 and *S. cerevisiae* Tof1, human Timeless is required for replication fork arrest at RFBs to coordinate the progression of replication with transcription activity in HeLa cells (Figure 2C) [65]. Thus, the role of the FPC at these pausing sites is conserved between yeast and mammalian cells. However, unlike in yeast, where replication pauses at the RFBs in a polar manner, replication in mammalian cells can be blocked in both directions [36,66].

The mechanism by which the FPC operates at RFB has begun to be elucidated. In *S. cerevisiae*, Tof1 and Csm3 are phosphorylated, and this phosphorylation promotes association of the FPC to the replication fork via interaction with the CMG helicase. At RFB sites, the FPC promotes stable fork arrest by antagonizing the Rrm3 helicase and thus preventing the removal of Fob1 [67,68,69]. In mammalian cells, the FPC inhibits the helicase activity of the CMG complex and the DNA-dependent ATPase activity of mini chromosome maintenance (Mcm) 2–7 proteins [70]. Although these studies are performed in vitro, it is possible that the FPC inhibits DNA helicase activity to promote fork arrest at programmed fork pausing sites throughout the chromosome. In addition to Timeless- and Tipin-related proteins, the FPC functions together with a third component, Claspin/Mrc1, which is required for activation of the inter-S phase checkpoint [71,72]. In *S. pombe*, Mrc1 is associated with FPC and plays a role in replication fork pausing at rDNA repeats, *MPS1* and *RTS1* at the mating-type locus, and *tRNA* genes. This function of *S*. *pombe* Mrc1 is mediated via a conserved helix-turn-helix DNA-binding domain that is also present in metazoan Claspin, but not in *S*. *cerevisiae* Mrc1 [73]. Future investigation is warranted to understand the molecular role of Claspin/Mrc1 in fork pausing.

Lastly, a recent study has suggested a role for Dicer (Dcr1) in replication fork arrest at rDNA loci. Dcr1, the enzyme that processes precursor RNAs into small interfering RNA (siRNA), is required for rDNA copy number maintenance [74]. Dcr1 regulates transcription termination and maintains genome stability at rDNA and other replication-pausing sites, such as protein-coding genes and transfer RNA genes (tRNAs). Collisions may occur between transcription and replication at these sites as represented by RNA Pol II enrichment in the absence of Dcr1. Such a role of Dcr1 in coordinating transcription and replication seems to be independent of its role in the RNAi pathway, as there is no RNA Pol II enrichment at these sites when mutations that abolish the canonical RNAi pathway were introduced [74]. Interestingly, *dcr1*∆ *swi3*∆ double deletion mutants show synthetic growth defects and hypersensitivity to replication-stressing agents [75], suggesting a role for Dicer in fork pausing at rDNA loci. Therefore, it appears that multiple pathways cooperate together to ensure fork pausing at rDNAs in order to preserve genomic integrity.

### 2.3. Centromeres

**Figure 3:**
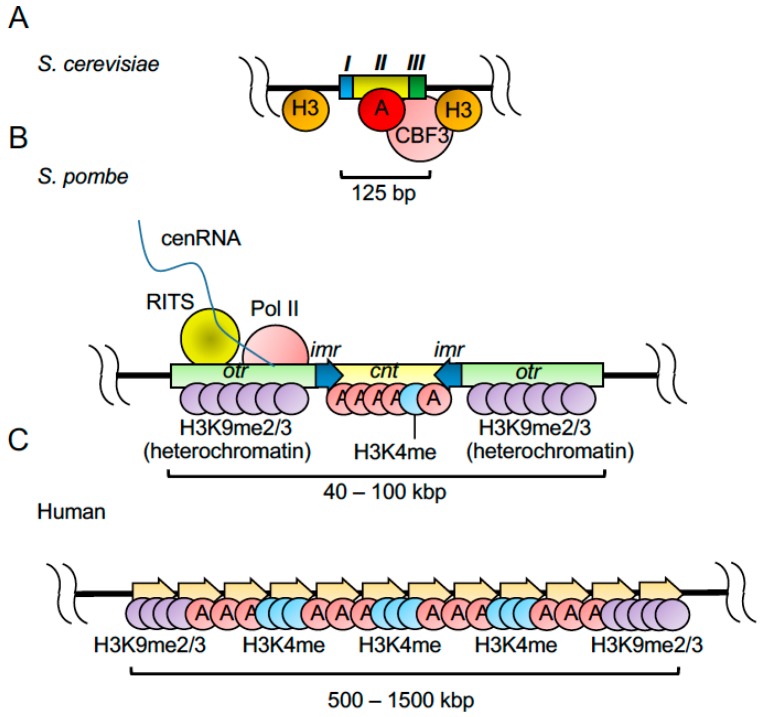
Centromere structures in *S. cerevisiae*, *S. pombe*, and humans. (**A**) Budding yeast has point centromeres, which are comprised of a 125 bp DNA sequence containing centromere DNA elements I (*CDEI*), II (*CDEII*), and III (*CDEIII*). The CBF3 complex binds to *CDEIII* and is involved in fork pausing at centromeres. (**B**) Fission yeast has regional centromeres, which consist of 40 to 100 kb DNA sequences including outer repeats (*otr*), inner repeats (*imr*), and the central core centromeric sequence (*cnt*). Pericentric heterochromatin at outer repeats presents histone H3 lysine 9 di- and tri-methylation and may cause fork pausing. RNAi-mediated silencing pathways are involved in releasing RNA polymerase II to maintain replication fork structure at centromeric regions. (**C**) Human centromeres contain alpha satellite repeats and recruit histone CENP-A. Other human centromere features include histone H3 lysine 9 di/tri-methylation and H3 lysine 4 mono-methylation.

Centromeres are large chromatin structures responsible for the proper segregation of chromosomes during mitosis and meiosis [76,77]. Defects in centromere regulation result in chromosome missegregation and aneuploidy [78,79]. Centromeres are characterized by an intricate structure that generates obstacles for DNA replication [80,81]. In most species, centromeres are organized into two domains; a pericentromeric heterochromatin region and a centromeric core defined by the presence of the centromere-specific histone, CENP-A (centromere protein A), where the kinetochore assembles (Figure 3). The kinetochore is a multi-protein complex that mediates the attachment of spindle microtubules to centromeres [78,82,83]. This configuration of centromeres, termed “regional”, is more common in mammals and other eukaryotic model organisms including *S. pombe*, *Drosophila Melanogaster*, *Arabidopsis thaliana*, *Neurospora crassa*, and *Oryza sativa* [84]. In contrast, *S. cerevisiae* centromeres are defined by an ~125-bp DNA sequence and termed “point centromeres” because of their simple organization (Figure 3A) [76]. Replication fork pausing was detected at centromeres of various chromosomes (*CEN1*, *CEN3*, *CEN4* and *CEN6*), thus the ability to promote fork pausing seems to be a general property of *S. cerevisiae* centromeres [80,85]. Replication barriers at centromeres differ from those at rDNA and the mating-type locus in the sense that they do not completely stop replication but mostly cause fork pausing [80]. Furthermore, centromeric barriers appear to be non-polar and thus are able to pause forks coming from both directions. Studies that looked at replication fork pausing at *CEN3* and *CEN4* showed that pausing at these sites is dependent on Tof1 but not Mrc1 [86]. In addition, it is suggested that fork pausing at centromeres is mediated by protein–DNA complexes that involve the centromere-binding factor CBF3 (Figure 3A) [80], which is required for kinetochore formation and proper chromosome segregation [87]. Consistently, in *Candida albicans*, the kinetochore functions as a replication barrier at the centromere [88]. In this organism, which is genetically related to *S. cerevisiae*, centromeres have a more complex organization than in *S. cerevisiae* and span approximately 3 kb on each chromosome. In *C. albicans*, fork stalling at centromeres decreases in the absence of Rad51 and Rad52, two main homologous recombination factors involved in fork restart. Failure in fork stalling is attributed to the defects in kinetochore assembly found in *rad51* and *rad52* mutant cells. Studies demonstrated that Rad51 and Rad52 promote the recruitment of CENP-A at the programmed fork-stalling sites at early replicating centromeres [88]. Therefore, it is straightforward to suggest that kinetochore structures present significant obstacles to replisome progression. However, whether the replication forks can pass through the DNA-protein complex or the complex disassembles before replication is not understood.

*S. pombe* has regional centromeres and their organization is similar to that of higher eukaryotes (Figure 3B,C) [76,89]. Replication forks appear to stall at *S. pombe* centromeres probably due to the presence of highly repetitive DNA sequences and heterochromatin marks, both considered to be difficult-to-replicate features [81,90]. Contrary to other heterochromatin regions including telomeres, centromeres in fission yeast replicate early in S phase [91]. The early replication of centromeres is linked to RNAi expression and heterochromatin assembly [92,93], and RNAi-mediated silencing pathways play a conserved role across species at heterochromatin regions and transposons [94,95]. Interestingly, the integrity of centromeres is maintained by its heterochromatin configuration and replication-fork-stabilizing factors that inhibit recombination at centromeres [96]. These findings suggest a role of heterochromatin and RNAi in replication fork pausing at centromeres (Figure 3B). Consistently, CENP-B (centromere protein B), which is associated with centromeric heterochromatin, is suggested to have a critical role in preservation of genomic integrity when forks are paused [47]. Furthermore, Zaratiegui et al. demonstrated that the RNAi machinery releases RNA Pol II from the DNA, allowing for the completion of DNA replication and preventing collisions between transcription and replication machineries [75]. Failure to release RNA Pol II causes fork stalling and promotes activation of homologous recombination pathways for repair. Furthermore, low concentrations of the replication inhibitor hydroxyurea that is innocuous to RNAi-deficient mutants, including *dcr1*∆, *ago1*∆ and *rdp1*∆ mutants, become highly toxic when the FPC component Swi3 was also deleted from these cells. Thus, stalled forks in the absence of the RNAi pathway are maintained by the FPC in order to prevent genomic instability [75]. Consistent with these results, in *Xenopus*, the Swi3 homolog Tipin is required for efficient replication of centromeric DNA and works together with Mta2, the activator subunit of the nucleosome remodeling and deacetylase complex (NuRD) to prevent fork reversal, most probably at difficult-to-replicate sites [97]. Further investigation is required to elucidate the details of how fork pausing is established at centromeres.

### 2.4. tRNA Genes and LTR Retrotransposons

Genetic screenings in *S. cerevisiae* identified numerous essential genes involved in preventing spontaneous DNA damage and genome rearrangements [98,99,100]. These include many DNA replication factors involved in different stages of DNA replication processes (initiation: *CDC45*, *DBF4*, *DPB11*, *MCM4*, *MCM5*, *MCM7*, and *PSF2*; elongation: *CDC45*, *DNA2*, *MCM4*, *MCM5*, *MCM7*, *POL2*, *POL30*, *PSF2*, *RFC2*, and *RFC5*; and termination: *UBC9*) [100]. Importantly, genome rearrangements were mapped to yeast fragile sites, including *Ty* retrotransposons, tRNA genes, early origins of replication, and replication termination sites [100]. These sites are prone to fork stalling, breakage, and chromosomal rearrangements, particularly, when DNA replication is compromised [101,102] or when checkpoint activation is defective [103,104,105].

Early work in budding yeast identified polar replication pause sites at *Ty1-LTR* and tRNA genes. These sites arrest forks moving in opposite direction to transcription [106] and may represent fragile sites when DNA replication is compromised, leading to genomic instability. Indeed, when the level of DNA polymerase α is reduced, chromosome translocations were greatly induced due to hyper-recombination at *Ty* elements [101].

Eukaryotic genomes contain a large number of tRNA genes, which are highly transcribed by RNA Pol III [107]. The program tRNAscan-SE [108] identified 186 and 286 tRNA genes in fission and budding yeast, respectively. The same program also identified 513 tRNAs in humans and 430 tRNAs in mouse [109]. In addition to their role in decoding mRNA sequences to proteins, tRNA genes also function in genome organization and stability [110]. tRNA genes and related RNA Pol III promoter elements can act as DNA replication barriers, as well as boundaries to separate different chromatin domains that comprise regulatory gene expression units [106,111,112]. Although this chromatin-boundary function has only been demonstrated in yeast, there is potential for these sites to play a similar role in mammalian cells as well [113].

In *S. cerevisiae*, fork pausing at tRNA genes requires Tof1 but not Mrc1, similar to what occurs at centromeres [86]. Active transcription of tRNA genes is also required for their replication-barrier activity [106]. Two hypotheses can explain the formation of these barriers; the first argues that supercoiling in the parental strand generated by the transcription and replication complexes causes a significant topological stress that prevents the progression of the replication fork. The second hypothesis proposes that barrier activity at these tRNA genes is a consequence of the direct interaction between replication and transcription machineries [106]. Although both hypotheses are not mutually exclusive, the exact nature of the replication barriers at tRNA genes is still subject of debate.

**Figure 4:**
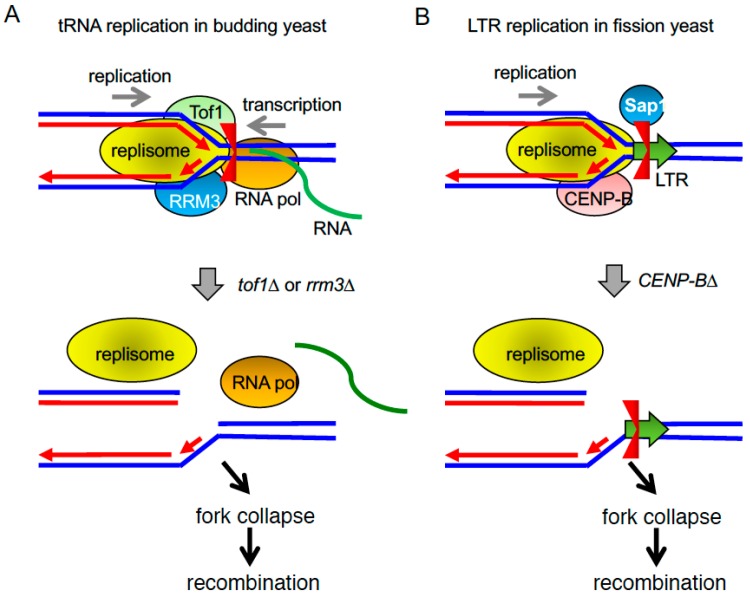
Replication fork pausing at tRNAs and LTRs. (**A**) The replication fork stalls at tRNA genes in a manner dependent on Tof1 in budding yeast. This fork stalling may prevent fork collapse due to collision between the replication and transcription machineries. Rrm3 sweepase appears to remove non-nucleosomal protein–DNA complex at the fork to facilitate fork progression at tRNA genes. (**B**) Polar fork pausing at LTRs is mediated by Sap1 in fission yeast. CENP-B-related proteins maintain fork stability at LTRs.

The replication barriers at tRNA genes may prevent collisions between replication and transcription machineries (Figure 4A). This idea is based on the studies that used mutations in the *S. cerevisiae* Rrm3 helicase, a member of the Pif1 DNA helicase family involved in genome maintenance [114]. *rrm3* mutations result in an increase in fork pausing and recombination potential at tRNA genes [85,103]. The elevated recombination appears to be dependent on collisions between RNA Pol III and the replication machinery in the absence of Rrm3, although the DNA sequences at the tRNA pausing sites themselves may not be highly recombinogenic [115]. tRNA pausing in *rrm3*∆ mutants is eliminated when the TFIIIC complex, required for transcription initiation, is removed from a tRNA gene [85]. Rrm3 appears to be a component of the replisome complex, providing a “sweepase” activity throughout the genome, in order to remove non-nucleosomal protein–DNA complexes ahead of the replication fork [116]. Such a sweepase function is conserved in *S. pombe* Pfh1, a Pif1-related DNA helicase [117]. Therefore, both protein–DNA complexes and transcription activity itself may contribute to the barrier or fork pausing activity at tRNA genes.

In *S. pombe*, tRNA genes can act as nonpolar replication fork barriers. About half of all 171 tRNA genes constitute fork pausing sites and bind to Pfh1, a helicase suggested to promote replication by displacing RNA Pol III from DNA during replication [118]. In contrast to rDNA fork barriers, tRNA barrier activity is independent of the FPC subunit Swi1. However, Swi1 still has an important role at tRNA genes; while in wild-type cells, the presence of tRNAs does not constitute a recombination hotspot, in *swi1*∆ cells, tRNAs become recombination hotspots and a source of genomic instability even though replication forks pause at tRNA genes at similar levels in both wild-type and *swi1*∆ cells [112]. It is noteworthy that the sequence homology between different tRNA copies and their clustered organization in the genome are thought to enhance the recombinogenic potential at tRNA loci [119]. Consistent with the role of Swi1 at tRNA genes, *swi1*∆ mutants show increased Rad3^ATR^-dependent H2A phosphorylation at tRNA loci, suggesting high rates of DNA damage and recombination events in the absence of the FPC [120]. Since fork pausing does not seem to correlate with the levels of recombination at tRNAs, the way by which Swi1 regulates fork stability at these sites might be different than that at other replication barriers [112]. In contrast, deletion of Tof1, *S. cerevisiae* Swi1 homolog, leads to the loss of fork pausing at tRNAs (Figure 4A). Fork pausing at tRNA genes is restored in *tof1*∆ *rrm3*∆ mutants [68,85], and similar interactions between Tof1–Csm3 and Rrm3 were found at the *Ter* sites in the rDNA [68]. Therefore, although *tRNA* genes and other RNA Pol III-transcribed elements can become recombinogenic targets when fork stability is impaired by loss of the FPC, there seems to be a fundamental difference in the establishment of fork pausing at tRNA genes between *S. pombe* and *S. cerevisiae*.

Retrotransposons are ubiquitously present in most genomes and they are involved in genome organization, function, and evolution [121]. These elements replicate via an RNA intermediate that converts into cDNA, which is then inserted along the genome. The fission yeast genome contains two types of LTR retrotransposons, Tf1 and Tf2 [122]. Recent studies revealed that Sap1, a DNA-binding factor that promotes fork pausing at rDNA loci, plays a critical role in targeting LTR retrotransposons to specific genome sites (Figure 4B) [123]. Both Tf1 and Tf2 are preferentially targeted to nucleosome free regions that coincide with RNA Pol II-transcribed gene promoters [124,125]. Interestingly, Sap1 is also identified as a *trans-*acting general regulatory factor that binds to nucleosome free regions and promotes nucleosome eviction [126]. Consistent with this finding, Sap1 tethers the Tf1 cDNA to Sap1-binding sites, thus guiding the insertion of the Tf1 transposons along the chromosome in a manner dependent on Sap1’s ability to arrest replication forks [123]. Tf2 LTR transposons in *S. pombe* function as polar replication barriers. Deletion of *abp1* and *cbh1*, two CENP-B homologs, causes recombination at these LTRs (Figure 4B), and this phenotype is suppressed in the *sap1-c* mutant in which Sap1 fails to bind to LTRs [47]. These results suggest that Abp1 and Cbh1 play a role in preventing genomic instability at LTRs (Figure 4B). Although the mechanisms by which CENP-B-related proteins regulate replication barriers are still elusive, Abp1 is reported to inhibit the expression of the transposons and neighboring genes by recruiting histone deacetylases (HDACs) [127]. Abp1 also affects expression of Tf1 and adjacent genes; Abp1 bound to Tf1 decreases transcription of adjacent stress genes, which might in turn prevent collisions with replication machinery and thus decrease recombination at the barrier [128]. Furthermore, Abp1 localizes to tRNA genes, suggesting a role of Abp1 in fork stability at tRNA genes [47]. Therefore, it is possible that CENP-B-related proteins regulate transcription of transposons and tRNAs, modulating replication barrier activity in order to prevent genomic instability at these loci.

Although there is no apparent Sap1 homolog, a similar mechanism of retrotransposon targeting at RFBs may be used in *S. cerevisiae*. For example, Ty1 retrotransposons are preferentially targeted at the 5′ region of Pol III-transcribed genes where replication forks are known to pause. However, unlike the case in *S. pombe*, Ty1 retrotransposons appear to be targeted to specific surfaces of the nucleosome and this integration process may be regulated by chromatin remodeling and modifying factors [129,130,131].

### 2.5. Fork Pausing and Termination at the Fission Yeast Mating-Type Locus

**Figure 5:**
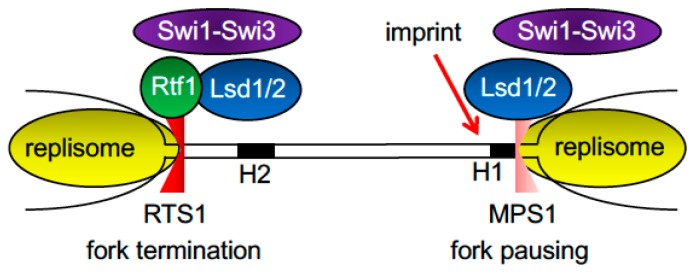
Fork pausing and termination at the fission yeast mating-type locus. Rtf1 binds to *RTS1* to prevent fork progression allowing the fork from the opposite direction to progress through the regions. This fork pauses at *MPS1* in order to generate an imprint required for recombination-mediated mating-type switching. Swi1–Swi3 and Lsd1/2 are both involved in fork stalling at RTS1 and *MPS1*.

Two of the most studied replication-fork block sites are involved in the process of mating-type switching in fission yeast. Each fission yeast cell carries one of two mating types, P or M, which depends on the allele (*mat2-P* or *mat3-M*, respectively) present at the mating-type (*mat1*) locus [4]. There are two FPC-dependent replication-block sites near *mat1*: the *mat1*-pausing site 1 (*MPS1*) and replication termination site 1 (*RTS1*) (Figure 5) [132]. As is the case for other programmed fork-pausing sites, a Myb/SANT-related protein is involved in mating-type switching. Rtf1, which has two Myb/SANT domains, directly binds to repeat DNA motifs at *RTS1* to block replisome progression (Figure 5) [133]. This strong polar replication block allows only one replication fork to move into the *mat1* locus. The moving fork pauses at *MPS1*, generating the essential imprint that initiates a replication-coupled recombination process, leading to the mating-type switch event [134,135]. Other *trans*-acting factors are necessary for mating-type switching; replication fork pausing at *MPS1* is dependent on Lsd1 and Lsd2 (Figure 5) [136], lysine-specific demethylases that are required for demethylation of histone H3 at its lysine 4 (H3K4) and lysine 9 (H3K9) residues for transcriptional regulation [137,138,139]. Lsd1 and Lsd2 appear to work upstream of the FPC to pause replication forks because recruitment of Swi1 at *MPS1* is significantly reduced in the *lsd1*-mutant [136]. Interestingly, Lsd1 and Lsd2 are also required at other FPC-mediated fork pausing sites including *RTS1* and RFBs at rDNA repeats, suggesting a role for either of these demethylases in FPC-dependent fork pausing. These findings may also suggest a role of histone modifications in replication-fork pausing at *MPS1*; however, Set1 and Clr4 methyltransferases, which specifically methylate H3K4 and H3K9, respectively, were not involved in the fork pausing [136]. Consistently, a non-enzymatic function of Lsd1 in chromatin regulation has also been suggested [140,141]. Future studies should determine if the role of Lsd1 and Lsd2 demethylases in fork pausing is exclusively enzymatic or if they also have a direct and structural role in FPC recruitment and fork pausing.

### 2.6. DNA Barriers Mediated by Repetitive DNA and Secondary Structures

**Figure 6:**
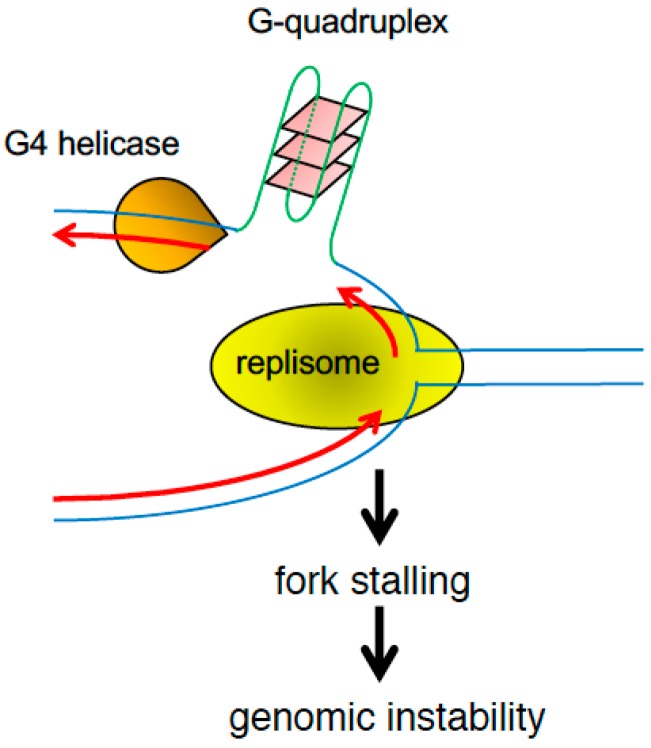
G-quadruplex structures form at a variety of genomic regions including telomeres and promoters and prevent fork progression leading to genomic instability.

Inverted repeats (IRs), mirror repeats (MRs), and direct tandem repeats (DTRs) are all common features of eukaryotic genomes that have potential to undergo structural transitions and generate secondary structures [142]. IRs can form cruciform structures in double-stranded DNA and hairpins in ssDNA, while MRs can assemble intramolecular triple-helices called H-DNA. DTRs can adopt a wide range of structures that depend on their base composition. The best-studied examples are G-quadruplexes, which are formed by tandem guanidines (Figure 6) [2]. Over two-dozen human hereditary disorders are caused by repeat expansions or contractions attributed to defects in DNA replication. While trinucleotide repeat instability is the cause of the majority of these diseases, including fragile X mental retardation [143], Huntington’s disease [144], and myotonic dystrophy [145], expansion of tetra, penta and dodecameric repeats are also linked to human diseases [146,147,148]. Therefore, understanding how cells achieve accurate replication of these structures is of importance.

DNA triplex structures form in vivo and cause replication fork pausing and genomic instability [149,150,151,152,153]. Although some reports suggest that triplex DNA can act as a replication obstacle [154,155,156,157], others propose that the unusual structure of the triplex DNA is recognized as DNA damage and processed by DNA repair proteins [158].

G-quadruplexes are stable DNA secondary structures that are formed by the stacking of groups of four guanidine residues within a single or multiple DNA strands and stabilized by Hoogsteen bonds (Figure 6) [159]. Although their formation in vivo is still under debate, G-quadruplex formation is favored by processes that open the double helix and expose ssDNA. Such processes include transcription and DNA replication, where G-quadruplexes can emerge at both leading and lagging strands [160]. Sequences that can potentially form G-quadruplexes in vitro, called G4 motifs, are highly prevalent in bacterial and eukaryotic genomes. In *S. pombe*, G4 motifs are enriched at telomeres, RNA Pol II-dependent promoters, and rDNA repeats, and such distribution of G4 motifs is conserved in *S. cerevisiae* and human genomes [161,162,163,164]. G-quadruplexes can stall prokaryote and eukaryote DNA polymerases in vitro [165,166] and are highly mutagenic in vivo [167]. Thus, G-quadruplexes could constitute physical barriers for the replication machinery [2], posing a serious threat to genome stability [160]. Consistently, in humans, fork stalling and genome instability associated with G4 motifs are linked to common translocation events associated with acute lymphoblastic leukemia [168].

G-quadruplex formation may be especially favored during lagging-strand replication, leading to genomic instability as replication forks may stall at these structures (Figure 6) [159]. G-quadruplexes were shown to hinder DNA synthesis by human DNA polymerase δ and several translesion polymerases [169]. Consistently, early studies demonstrated that, in *E. coli*, repeat loss occurs preferentially during lagging-strand synthesis [170]. Nonetheless, formation of G-quadruplexes can also occur in the leading strand and create instability, in particular, when these structures are stabilized by Phen-DC3 or introduced in a helicase-deficient *pif1*∆ background [160].

Several classes of DNA helicases are involved in resolution of G-quadruplexes, and mutations in many of these helicases are known to cause human diseases associated with genomic instability. One class of DNA helicases involved in G4-unwinding contains an iron-sulphur (Fe-S) cluster involved in accepting and donating electrons [171]. One such helicase, FANCJ (Fanconi anemia complementation group J), is involved in the Fanconi anemia (FA) DNA repair pathway and is required for the repair of interstrand crosslinks [172]. FANCJ unwinds G-quadruplexes in the context of telomeric- and triplet-repeat DNA sequences in vitro. This activity is inhibited by a telomestatin that specifically binds G-quadruplexes [173]. Telomestatin also inhibits growth and induces DNA damage in FANCD2-deficient human cells, suggesting a role for FANCJ in unwinding G-quadruplexes in vivo [173,174]. Consistently, fork stalling occurs at a higher rate in FANCJ-deleted avian DT40 cells, suggesting that FANCJ is required for efficient replication through G-quadruplexes. Defects in the FA pathway are associated with bone marrow failure and a strong predisposition to cancer [172], although FANCJ DNA helicase appears to unwind G-quadruplexes independently of the FA pathway [173,175].

Another class of G4-unwinding helicases includes RecQ-related helicases WRN (Werner’s syndrome) and BLM, whose mutations cause cancer susceptible disorders Werner and Bloom syndromes, respectively [171]. These helicases have G4-unwinding activity in vitro and facilitate DNA replication through G-quadruplexes at telomeres [171,176]. These helicases unwind duplexes in the 3′–5′ direction, which is the opposite polarity to FANCJ-mediated unwinding activity. Interestingly, BLM interacts directly with FANCJ, and together, these helicases unwind a damaged DNA substrate more efficiently than either single helicase [177]. Furthermore, FANCJ functions in concert with both BLM and WRN to maintain epigenetic stability at a G-quadruplex-containing locus, suggesting that these helicases remove G-quadruplex from opposite directions [178].

Finally, the Pif1-related helicases have robust G-quadruplex unwinding activity. Using purified proteins, the Zakian group showed that budding yeast Pif1 preferentially binds and unwind G-quadruplex DNA. Strikingly, Pif1 was much more efficient in G-quadruplex unwinding than human WRN, *E. coli* RecQ, and Sgs1 (budding yeast RecQ) [27]. In *S. pombe*, Pfh1, a Pif1-related helicase, is preferentially recruited to regions with G4 motifs and unwinds G-quadruplex structures. In the absence of Pfh1, replication forks pause at G-quadruplexes, leading to DNA damage and genome instability [28]. A recent paper shows that, both telomeric and rDNA sequences from *S. pombe*, can form G-quadruplexes in vitro and that Pfh1 is able to unwind these structures [179]. Interestingly, a study suggested that G-quadruplexes not only pose replicative obstacles but also function as regulatory elements that aid in lagging-strand synthesis [180], and emerging evidence suggest the role as *cis*-acting regulatory elements of G-quadruplexes in DNA replication as well as in transcription, translation, and telomere maintenance [181]. Interestingly, G-quadruplexes are extensively found near transcriptional start sites (TSS). Such DNA secondary structures at TSS may affect DNA topology, creating a dynamic equilibrium between duplex DNA and secondary conformation, in order to not only regulate transcription [182,183], but also control replication initiation [184,185,186]. Further studies are necessary to understand the mechanisms by which G-quadruplex DNA regulates multiple cellular processes.

## 3. Coordination between Transcription and Replication Machineries

DNA replication and gene transcription are fundamental genetic processes required for cell growth and division. Both processes are carried out by large protein complexes that move processively along the genome and cause temporary but significant alterations to the DNA structure. Collisions between the transcription and replication machineries are a clear source of genomic instability in both prokaryotes and eukaryotes [187,188,189], and have recently been linked to oncogene-induced DNA damage in cancer cells [190]. In this section, we attempt to summarize the current knowledge on the molecular basis of transcription–replication encounters and the consequences of their dysregulation.

### 3.1. Transcription–Replication Encounters

**Figure 7:**
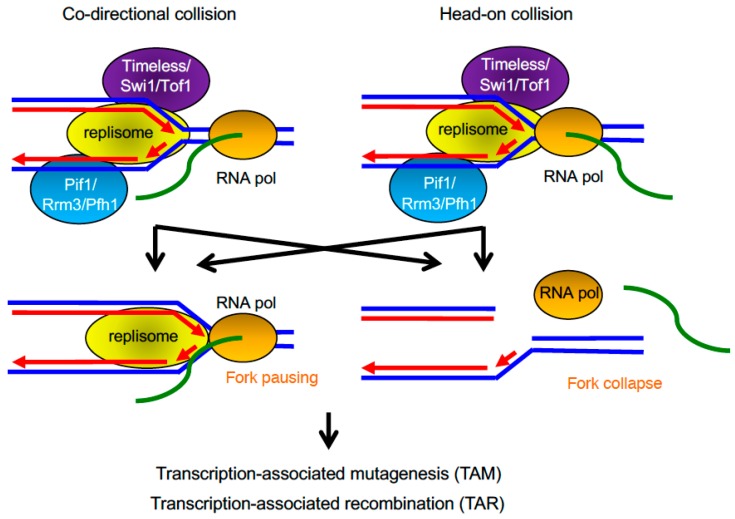
The replication and transcription machinery share the same template DNA, leading to collisions between the two. Timeless-related proteins may promote fork pausing, while Pif1-related DNA helicase facilitate fork progression through highly transcribed regions. Deregulation of fork maintenance at highly transcribed genes results in TAM and TAR.

Transcription–replication encounters can occur when the transcription and the replication machineries move in the opposite direction and converge upon each other (head-on collision). Because these machineries have different velocities, collision also occurs when the two machineries move in the same direction (co-directional collision) (Figure 7). Studies in *E. coli* and *S. cerevisiae* show that transcription impedes replisome progression, resulting in the arrest of replication forks [191,192,193]. Activation of DNA damage response pathways, elevated mutagenesis, and chromosomal instability at actively transcribed loci suggest that replication forks also collapse upon encountering the transcription machinery (Figure 7) [194,195].

Head-on collisions are thought to be more detrimental than co-directional collisions. The effect of transcription directionality on fork progression was initially studied in bacteria using inverted ribosomal RNA operons that are naturally transcribed in a co-directional fashion with replication [196]. This and other studies in *E. coli* and *B. subtilis* demonstrated that replication slows down when transcription units are arranged in a head-on direction with respect to fork movement, as compared to transcription units arranged in a co-directional manner [192,196,197]. Head-on collisions were also shown to generate positive supercoiling that could lead to fork reversal and the formation of chicken-foot structures [198]. Furthermore, large-scale genome organization studies in both prokaryotes and eukaryotes show that genes are positioned so that their transcription is co-directional with replication fork movement. This organization tends to be more pronounced near origins of replication and weakens as the distance to the origins increases [188,199,200].

Spatiotemporal separation between transcription and replication may be another evolutionary solution to prevent collisions. Although bacteria species lack temporal separation [2], eukaryotic replication and transcription occur within spatially and temporally separated domains [201]. Although the majority of transcriptional activity in eukaryotes occurs during G1 phase, there are transcriptionally active loci in S phase, and these loci seem to be spatially separated from replicating regions [202]. Transcription and replication of rRNA genes (rDNA) in mammalian cells are an excellent example of such coordination [203,204]. Each rDNA locus undergoes a temporal sequence of reprogramming from active transcription to active replication. Following replication, the rDNA loci are reprogrammed for transcription [205]. In terms of spatial separation, actively transcribed rRNA genes are exclusively localized in the interior of the nucleolus [206,207], and replicating loci seem to be physically separated from their transcription by fibrillar centers that provide a structural barrier between domains [203]. In *S. cerevisiae*, actively transcribed genes localize near nuclear pores [208,209]. Although this may alleviate transcription–replication encounters by spatially separating transcription and replication activities, such genome reorganization can increase torsional stress in DNA associated with active transcription, causing negative consequences to both transcription and replication processes.

### 3.2. Highly Transcribed Regions as Replicative Obstacles

Studies suggest that replication forks pause during head-on encounters with the transcription machinery but only collapse in the presence of RNA polymerase arrays at highly transcribed operons [188]. Transcription-dependent fork pausing was reported in *E. coli,* both in vitro using phage components [191,210] and in vivo on plasmids [192], and in a chromosomal context [211]. In eukaryotes, a genome-wide analysis of DNA polymerase pause sites was performed in *S. cerevisiae*. This study demonstrated that highly transcribed RNA Pol II-dependent genes were significantly represented as replication pausing sites [211]. In fission yeast, replication fork pausing was also linked to increased recombination at the *leu2* locus, which is transcribed by RNA Pol II [193]. Further investigation suggests that stalled replication is a prerequisite to hyper-recombination [212]. Null mutants of *S. cerevisiae* Hpr1, a component of the THO complex, exhibit hyperrecombination phenotypes in addition to defects in transcriptional elongation and mRNA export to the cytoplasm [213,214,215,216,217,218]. However, the *hpr1-101* allele, which contains a point mutation in the *hpr1* gene, fails to cause hyperrecombination phenotypes although transcriptional elongation and mRNA export are inhibited in the mutant. This loss of the hyperrecombination phenotype is correlated with the absence of replication fork blockage in *hpr1* mutants, suggesting that hyperrecombination is caused by stalled replication forks [212]. Although the molecular mechanisms involved in replication pausing at transcription sites still remain unclear, DNA polymerase ε, a major replicative enzyme, is enriched throughout open reading frames in *S. cerevisiae*. Therefore, instead of promoter-associated protein complexes, the transcription machinery itself and/or nascent RNA seem to be the cause of replication fork pausing [211].

Studies in *E. coli* and *B. subtilis* have found that the intensity of the fork-arresting signal is correlated with the rate of transcription [192,196,197]. Highly transcribed genes impede replication when they are placed head-on to the replication fork, explaining why most highly expressed operons in bacteria are arranged in a co-directional orientation with respect to the direction of replication in the genome [199]. In eukaryotes, collisions at highly transcribed genes are blocked by DNA–protein barriers, as described above for rDNA repeats [219]. In addition to the rate of transcription, gene length also seems to play a role in genomic stability. Inverting long genes enhances the mutation rate in *B. subtilis*, suggesting that the co-directionality of long transcriptional units with replication prevents fork arrest and/or collapse in bacteria [220]. Consistently, many common fragile-sites (CFSs) in cancer cells co-localize with very large genes in human cells [221,222].

Several replication factors have been shown to play a specific role during replication of highly transcribed regions. The *S. pombe* Pfh1 helicase is required for efficient fork movement at highly transcribed RNA polymerase II-dependent genes and at other difficult-to-replicate regions such as rDNA loci. Because cells depleted of Phf1 are unviable in the absence of Swi1, accumulated stalled forks in the Pfh1 mutant cells may need to be stabilized by Swi1 for survival [223]. In relation to this idea, in budding yeast, roles of the Swi1/Timeless homolog, Tof1, and its partner, Csm3, were investigated for replisome protection at a RNA Pol III-dependent transcription site in a plasmid. Replication fork pausing is greatly attenuated in the *tof1*∆ mutant and significantly enhanced in the *rrm3*∆ mutant. Rrm3 is a member of the Pif1 family of helicases in budding yeast. Deletion of both *tof1* and *rrm3*, restores pausing to a level significantly higher than that of the wild-type cells [68]. Furthermore, Timeless–Tipin homologs in *S. cerevisiae, S. pombe*, and humans control the RFBs of rDNA genes in order to prevent collisions between transcription and replication complexes during ribosomal gene transcription [39,65,68,224], suggesting the general role of Timeless–Tipin in preventing genome instability at the interface of DNA replication and transcription (Figure 7).

### 3.3. Transcription-Associated Mutagenesis and Recombination

Transcription increases spontaneous and chemically induced mutations and also stimulates recombination. These events have been referred to as transcription-associated mutagenesis (TAM) and transcription-associated recombination (TAR), respectively (Figure 7) [193]. These mechanisms are conserved from bacteria to mammalian cells, and they both can be induced by transcription–replication collisions among other causes.

TAM can arise in a replication-dependent or -independent manner [225]. Although there is no strong evidence linking replication fork direction and TAM in higher eukaryotes, a higher rate of mutagenesis was reported in *B. subtilis* when transcription occurred in a head-on versus co-directional orientation [188]. In budding yeast, mutation rates are directly proportional to transcription levels; however, reversing the direction of replication subtly affects the occurrence of TAM [195]. Although the reason why head-on conflicts are more mutagenic than co-directional conflicts is not known, studies in yeast suggest that recombination might play a role. In particular, head-on transcription–replication collisions stimulate recombination more than co-directional encounters [226], and recombination-associated DNA synthesis appears to be an error-prone pathway [227,228].

**Figure 8:**
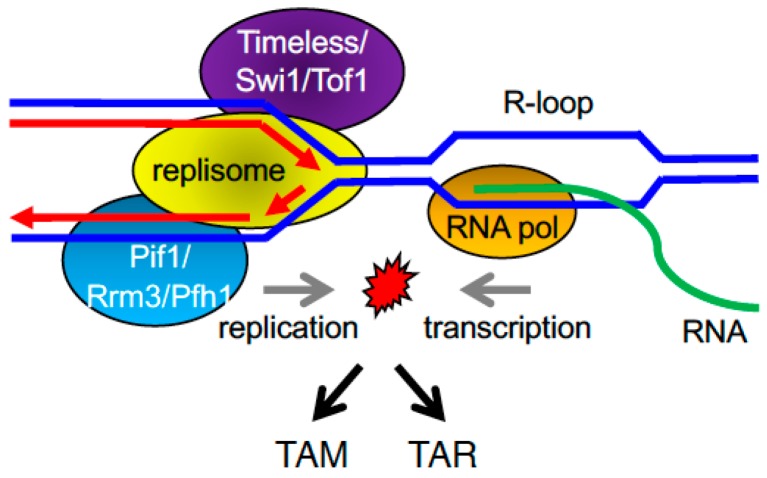
R-loops formed at the interface between transcription and replication may induce TAM and TAR. Proteins associated with the replisome may play important roles in minimizing R-loop formations to prevent genomic instability.

Although TAR is a very prevalent process in all organisms, its mechanisms remain unclear. One of the first reports on the effect of transcription on genetic stability in a eukaryotic system showed that HOT1, a segment of the ribosomal DNA locus that is actively transcribed by RNA polymerase I, could function as a *cis*-acting enhancer of recombination in *S. cerevisiae* [229]. A similar increase in recombination and spontaneous mutations was observed with high transcription levels by RNA Pol II [230,231]. Two different hypotheses explain the stimulation of spontaneous recombination by transcription: the first one centers around the increased accessibility of homologous recombination proteins to the DNA during transcription; the second hypothesis suggests that collisions between the transcription and replication machineries, the presence of stalled replication forks during transcription, or the formation of transcription-associated DNA:RNA hybrids (R-loops) can be significant sources of DNA damage and TAR (Figure 8). R-loops are three-strand RNA:DNA hybrid structures, where the nascent RNA hybridizes with the DNA template and causes the nontemplate strand to remain as ssDNA. R-loop formation and stabilization impair transcriptional elongation [232], and the stalled transcription machinery may be more prone to replication fork stalling, inducing TAR (Figure 8) [213].

The accessibility hypothesis is supported by experiments done in yeast treated with DNA-damaging agents. In this setting, a synergistic effect on recombination was observed between treatment with DNA-damaging agents and induction of transcription in a plasmid system where transcription can be induced by the *GAL1*- or the *tet*-regulated promoters. These results suggest that TAR induced by DNA-damaging agents may be, to a large extent, caused by the increased accessibility to the DNA that the DNA-damaging agents have during transcription [233]. Other structures formed during transcription, such as transcription-induced supercoiling and chromatin remodeling, may also promote homologous recombination by bringing homologous regions closer together [234,235]. Furthermore, negatively supercoiled DNA favors the formation of R-loops with the nascent mRNA, generating a stretch of ssDNA on the non-transcribed strand, which becomes more susceptible to DNA damage and recombination [213].

The collision hypothesis, on the other hand, suggests that transcription and replication occurring on the same DNA template can obstruct each other, resulting in stalled or collapsed replication forks that create templates for TAR (Figure 7). A central factor that affects replication progression is the formation of R-loops during transcription (Figure 8) [236]. Since R-loops can impair DNA integrity, multiple mechanisms exist to prevent and resolve R-loop structures: co-transcriptional assembly of RNP particles on the nascent RNA prevents the formation of R-loops from bacteria to metazoans; and the presence of nucleosomes prevents invasion of the RNA strand after passage of the transcription machinery in yeast and higher eukaryotes. In addition, RNA processing factors that assemble at the nascent RNA also prevent the accumulation of R-loops. Thus, mutants defective for these pathways, including transcription elongation [232], RNA splicing [237], and mRNA export [238] display genomic instability and elevated TAR.

## 4. Conclusions

Cells have developed a myriad of mechanisms to ensure error-free, stable, and processive DNA replication. These mechanisms include fork protection proteins that stabilize the fork when it stalls, checkpoint pathways that monitor fork stalling and delay cell cycle progression, helicases that remove DNA bound proteins ahead of the fork, and topoisomerases that release torsion and topological entanglements. In this setting, although initially counterintuitive, there are intrinsic regions across the genome that promote fork stalling. These genome regions have a central role in genome stability; however, how they function together with the replisome to promote genome stability is not completely clear. In this review, we aimed at presenting our current understanding of how cells deal with some of the replication obstacles present along the genome. We also intended to provide a clearer view of why these obstacles are retained throughout evolution as they also carry inherent regulatory functions, which we are starting to understand. Future studies are warranted to investigate the molecular function of replication fork blockage and its effects in genome stability and instability. Such investigations at the genome-wide level using multiple organisms may lead us to better understanding of the impact of fork stalling on genome maintenance. They will also help us elucidate how DNA replication and transcription processes are coordinated not only to preserve genome integrity but also to promote genome evolution.

## Supplementary Materials

The following are available online at www.mdpi.com/2073-4425/8/3/98/s1, Table S1: Replication barriers in human, *S. pombe*, and *S. cerevisiae* genomes.

## Abbreviations

The following abbreviations are used in this manuscript:

CBF3	centromere binding factor 3
CENP-A	centromere protein-A
CENP-B	centromere protein-B DSB: Double strand break
CMG	Cdc45-MCM-GINS
DTR	direct tandem repeat
FA	Fanconi Anemia
FPC	fork protection complex
IR	inverted repeat
LTR	long terminal repeat
MPS1	*mat1* pausing site 1
MR	mirror repeat
RFB	replication fork barrier
RNA Pol II	RNA polymerase II
RNA Pol III	RNA polymerase III
RTS1	replication termination site 1
ssDNA	single-stranded DNA
TAM	transcription-associated mutagenesis
TAR	transcription-associated recombination
TTF-1	transcription termination factor-I
TTS	transcription termination site

## References

[1] Lindahl T. (1993). Instability and decay of the primary structure of DNA. Nature.

[2] Mirkin E.V., Mirkin S.M. (2007). Replication fork stalling at natural impediments. Microbiol. Mol. Biol. Rev..

[3] Lambert S., Carr A.M. (2013). Replication stress and genome rearrangements: Lessons from yeast models. Curr. Opin. Genet. Dev..

[4] Egel R. (2005). Fission yeast mating-type switching: Programmed damage and repair. DNA Repair.

[5] Aguilera A., Gaillard H. (2014). Transcription and recombination: When RNA meets DNA. Cold Spring Harb. Perspect. Biol..

[6] Branzei D., Foiani M. (2010). Maintaining genome stability at the replication fork. Nat. Rev. Mol. Cell Biol..

[7] Miller K.M., Rog O., Cooper J.P. (2006). Semi-conservative DNA replication through telomeres requires Taz1. Nature.

[8] Segurado M., de Luis A., Antequera F. (2003). Genome-wide distribution of DNA replication origins at A+T-rich islands in *Schizosaccharomyces pombe*. EMBO Rep..

[9] Drosopoulos W.C., Kosiyatrakul S.T., Yan Z., Calderano S.G., Schildkraut C.L. (2012). Human telomeres replicate using chromosome-specific, rather than universal, replication programs. J. Cell Biol..

[10] Kurth I., Gautier J. (2010). Origin-dependent initiation of DNA replication within telomeric sequences. Nucleic Acids Res..

[11] Sfeir A., Kosiyatrakul S.T., Hockemeyer D., MacRae S.L., Karlseder J., Schildkraut C.L., de Lange T. (2009). Mammalian telomeres resemble fragile sites and require TRF1 for efficient replication. Cell.

[12] Watson J.D. (1972). Origin of concatemeric T7 DNA. Nat. New Biol..

[13] Ivessa A.S., Zhou J.Q., Schulz V.P., Monson E.K., Zakian V.A. (2002). *Saccharomyces* Rrm3p, a 5′ to 3′ DNA helicase that promotes replication fork progression through telomeric and subtelomeric DNA. Genes Dev..

[14] Gadaleta M.C., Das M.M., Tanizawa H., Chang Y.T., Noma K., Nakamura T.M., Noguchi E. (2016). Swi1^timeless^ prevents repeat instability at fission yeast telomeres. PLoS Genet..

[15] Leman A.R., Noguchi E. (2012). Local and global functions of Timeless and Tipin in replication fork protection. Cell Cycle.

[16] Noguchi E., Noguchi C., McDonald W.H., Yates J.R., Russell P. (2004). Swi1 and Swi3 are components of a replication fork protection complex in fission yeast. Mol. Cell. Biol..

[17] Gadaleta M.C., González-Medina A., Noguchi E. (2016). Timeless protection of telomeres. Curr. Genet..

[18] Urtishak K.A., Smith K.D., Chanoux R.A., Greenberg R.A., Johnson F.B., Brown E.J. (2009). Timeless maintains genomic stability and suppresses sister chromatid exchange during unperturbed DNA replication. J. Biol. Chem..

[19] Leman A.R., Dheekollu J., Deng Z., Lee S.W., Das M.M., Lieberman P.M., Noguchi E. (2012). Timeless preserves telomere length by promoting efficient DNA replication through human telomeres. Cell Cycle.

[20] Cooper J.P., Nimmo E.R., Allshire R.C., Cech T.R. (1997). Regulation of telomere length and function by a Myb-domain protein in fission yeast. Nature.

[21] Ohki R., Ishikawa F. (2004). Telomere-bound TRF1 and TRF2 stall the replication fork at telomeric repeats. Nucleic Acids Res..

[22] Ishikawa F. (2013). Portrait of replication stress viewed from telomeres. Cancer Sci..

[23] Makovets S., Herskowitz I., Blackburn E.H. (2004). Anatomy and dynamics of DNA replication fork movement in yeast telomeric regions. Mol. Cell. Biol..

[24] Sridhar A., Kedziora S., Donaldson A.D. (2014). At short telomeres Tel1 directs early replication and phosphorylates Rif1. PLoS Genet..

[25] Zimmermann M., Kibe T., Kabir S., de Lange T. (2014). TRF1 negotiates ttaggg repeat-associated replication problems by recruiting the BLM helicase and the TPP1/POT1 repressor of ATR signaling. Genes Dev..

[26] Hou X.M., Wu W.Q., Duan X.L., Liu N.N., Li H.H., Fu J., Dou S.X., Li M., Xi X.G. (2015). Molecular mechanism of G-quadruplex unwinding helicase: Sequential and repetitive unfolding of G-quadruplex by Pif1 helicase. Biochem. J..

[27] Paeschke K., Bochman M.L., Garcia P.D., Cejka P., Friedman K.L., Kowalczykowski S.C., Zakian V.A. (2013). Pif1 family helicases suppress genome instability at G-quadruplex motifs. Nature.

[28] Sabouri N., Capra J.A., Zakian V.A. (2014). The essential *Schizosaccharomyces pombe* Pfh1 DNA helicase promotes fork movement past G-quadruplex motifs to prevent DNA damage. BMC Biol..

[29] Lopez-Estrano C., Schvartzman J.B., Krimer D.B., Hernandez P. (1999). Characterization of the pea rDNA replication fork barrier: Putative cis-acting and trans-acting factors. Plant Mol. Biol..

[30] Hernandez P., Martin-Parras L., Martinez-Robles M.L., Schvartzman J.B. (1993). Conserved features in the mode of replication of eukaryotic ribosomal RNA genes. EMBO J..

[31] MacAlpine D.M., Zhang Z., Kapler G.M. (1997). Type I elements mediate replication fork pausing at conserved upstream sites in the tetrahymena thermophila ribosomal DNA minichromosome. Mol. Cell. Biol..

[32] Hyrien O., Mechali M. (1993). Chromosomal replication initiates and terminates at random sequences but at regular intervals in the ribosomal DNA of *Xenopus* early embryos. EMBO J..

[33] Lopez-estrano C., Schvartzman J.B., Krimer D.B., Hernandez P. (1998). Co-localization of polar replication fork barriers and rRNA transcription terminators in mouse rDNA. J. Mol. Biol..

[34] Brewer B.J., Fangman W.L. (1988). A replication fork barrier at the 3′ end of yeast ribosomal RNA genes. Cell.

[35] Linskens M.H., Huberman J.A. (1988). Organization of replication of ribosomal DNA in *Saccharomyces cerevisiae*. Mol. Cell. Biol..

[36] Little R.D., Platt T.H., Schildkraut C.L. (1993). Initiation and termination of DNA replication in human rRNA genes. Mol. Cell. Biol..

[37] Gerber J.K., Gogel E., Berger C., Wallisch M., Muller F., Grummt I., Grummt F. (1997). Termination of mammalian rDNA replication: Polar arrest of replication fork movement by transcription termination factor TTF-I. Cell.

[38] Sanchez J.A., Kim S.M., Huberman J.A. (1998). Ribosomal DNA replication in the fission yeast, *Schizosaccharomyces pombe*. Exp. Cell Res..

[39] Krings G., Bastia D. (2004). *swi1*- and *swi3*-dependent and independent replication fork arrest at the ribosomal DNA of *Schizosaccharomyces pombe*. Proc. Natl. Acad. Sci. USA.

[40] Sanchez-Gorostiaga A., Lopez-Estrano C., Krimer D.B., Schvartzman J.B., Hernandez P. (2004). Transcription termination factor Reb1p causes two replication fork barriers at its cognate sites in fission yeast ribosomal DNA in vivo. Mol. Cell. Biol..

[41] Krings G., Bastia D. (2005). Sap1p binds to *Ter1* at the ribosomal DNA of *Schizosaccharomyces pombe* and causes polar replication fork arrest. J. Biol. Chem..

[42] Arcangioli B., Klar A.J. (1991). A novel switch-activating site (SAS1) and its cognate binding factor (SAP1) required for efficient *mat1* switching in *Schizosaccharomyces pombe*. EMBO J..

[43] Ghazvini M., Ribes V., Arcangioli B. (1995). The essential DNA-binding protein Sap1 of *schizosaccharomyces pombe* contains two independent oligomerization interfaces that dictate the relative orientation of the DNA-binding domain. Mol. Cell. Biol..

[44] de Lahondes R., Ribes V., Arcangioli B. (2003). Fission yeast Sap1 protein is essential for chromosome stability. Eukaryot Cell.

[45] Noguchi C., Noguchi E. (2007). Sap1 promotes the association of the replication fork protection complex with chromatin and is involved in the replication checkpoint in *Schizosaccharomyces pombe*. Genetics.

[46] Mejia-Ramirez E., Sanchez-Gorostiaga A., Krimer D.B., Schvartzman J.B., Hernandez P. (2005). The mating type switch-activating protein Sap1 is required for replication fork arrest at the rRNA genes of fission yeast. Mol. Cell. Biol..

[47] Zaratiegui M., Vaughn M.W., Irvine D.V., Goto D., Watt S., Bahler J., Arcangioli B., Martienssen R.A. (2011). CENP-B preserves genome integrity at replication forks paused by retrotransposon LTR. Nature.

[48] Krings G., Bastia D. (2006). Molecular architecture of a eukaryotic DNA replication terminus-terminator protein complex. Mol. Cell. Biol..

[49] Singh S.K., Sabatinos S., Forsburg S., Bastia D. (2010). Regulation of replication termination by Reb1 protein-mediated action at a distance. Cell.

[50] Biswas S., Bastia D. (2008). Mechanistic insights into replication termination as revealed by investigations of the Reb1-*Ter3* complex of *Schizosaccharomyces pombe*. Mol. Cell. Biol..

[51] Melekhovets Y.F., Shwed P.S., Nazar R.N. (1997). In vivo analyses of RNA polymerase I termination in *Schizosaccharomyces pombe*. Nucleic Acids Res..

[52] Bastia D., Singh S.K. (2011). “Chromosome kissing” and modulation of replication termination. Bioarchitecture.

[53] Rodriguez-Sanchez L., Rodriguez-Lopez M., Garcia Z., Tenorio-Gomez M., Schvartzman J.B., Krimer D.B., Hernandez P. (2011). The fission yeast rDNA-binding protein Reb1 regulates G1 phase under nutritional stress. J. Cell Sci..

[54] Choudhury M., Zaman S., Jiang J.C., Jazwinski S.M., Bastia D. (2015). Mechanism of regulation of ‘chromosome kissing’ induced by Fob1 and its physiological significance. Genes Dev..

[55] Huang J., Moazed D. (2003). Association of the RENT complex with nontranscribed and coding regions of rDNA and a regional requirement for the replication fork block protein Fob1 in rDNA silencing. Genes Dev..

[56] Shou W., Seol J.H., Shevchenko A., Baskerville C., Moazed D., Chen Z.W., Jang J., Shevchenko A., Charbonneau H., Deshaies R.J. (1999). Exit from mitosis is triggered by Tem1-dependent release of the protein phosphatase Cdc14 from nucleolar RENT complex. Cell.

[57] Straight A.F., Shou W., Dowd G.J., Turck C.W., Deshaies R.J., Johnson A.D., Moazed D. (1999). Net1, a Sir2-associated nucleolar protein required for rDNA silencing and nucleolar integrity. Cell.

[58] Kobayashi T., Horiuchi T., Tongaonkar P., Vu L., Nomura M. (2004). Sir2 regulates recombination between different rDNA repeats, but not recombination within individual rRNA genes in yeast. Cell.

[59] Kobayashi T., Ganley A.R. (2005). Recombination regulation by transcription-induced cohesin dissociation in rDNA repeats. Science.

[60] Bairwa N.K., Zzaman S., Mohanty B.K., Bastia D. (2010). Replication fork arrest and rDNA silencing are two independent and separable functions of the replication terminator protein Fob1 of *Saccharomyces cerevisiae*. J. Biol. Chem..

[61] Jakociunas T., Domange Jordo M., Ait Mebarek M., Bunner C.M., Verhein-Hansen J., Oddershede L.B., Thon G. (2013). Subnuclear relocalization and silencing of a chromosomal region by an ectopic ribosomal DNA repeat. Proc. Natl. Acad. Sci. USA.

[62] Grummt I., Maier U., Ohrlein A., Hassouna N., Bachellerie J.P. (1985). Transcription of mouse rDNA terminates downstream of the 3′ end of 28S RNA and involves interaction of factors with repeated sequences in the 3′ spacer. Cell.

[63] Bartsch I., Schoneberg C., Grummt I. (1987). Evolutionary changes of sequences and factors that direct transcription termination of human and mouse ribsomal genes. Mol. Cell. Biol..

[64] Grummt I., Rosenbauer H., Niedermeyer I., Maier U., Ohrlein A. (1986). A repeated 18 bp sequence motif in the mouse rDNA spacer mediates binding of a nuclear factor and transcription termination. Cell.

[65] Akamatsu Y., Kobayashi T. (2015). The human RNA polymerase I transcription terminator complex acts as a replication fork barrier that coordinates the progress of replication with rRNA transcription activity. Mol. Cell. Biol..

[66] Lebofsky R., Bensimon A. (2005). DNA replication origin plasticity and perturbed fork progression in human inverted repeats. Mol. Cell. Biol..

[67] Bastia D., Srivastava P., Zaman S., Choudhury M., Mohanty B.K., Bacal J., Langston L.D., Pasero P., O’Donnell M.E. (2016). Phosphorylation of CMG helicase and Tof1 is required for programmed fork arrest. Proc. Natl. Acad. Sci. USA.

[68] Mohanty B.K., Bairwa N.K., Bastia D. (2006). The Tof1p-Csm3p protein complex counteracts the Rrm3p helicase to control replication termination of *Saccharomyces cerevisiae*. Proc. Natl. Acad. Sci. USA.

[69] Mohanty B.K., Bairwa N.K., Bastia D. (2009). Contrasting roles of checkpoint proteins as recombination modulators at Fob1-Ter complexes with or without fork arrest. Eukaryot Cell.

[70] Cho W.H., Kang Y.H., An Y.Y., Tappin I., Hurwitz J., Lee J.K. (2013). Human Tim-Tipin complex affects the biochemical properties of the replicative DNA helicase and DNA polymerases. Proc. Natl. Acad. Sci. USA.

[71] Calzada A., Hodgson B., Kanemaki M., Bueno A., Labib K. (2005). Molecular anatomy and regulation of a stable replisome at a paused eukaryotic DNA replication fork. Genes Dev..

[72] Katou Y., Kanoh Y., Bando M., Noguchi H., Tanaka H., Ashikari T., Sugimoto K., Shirahige K. (2003). S-phase checkpoint proteins Tof1 and Mrc1 form a stable replication-pausing complex. Nature.

[73] Zech J., Godfrey E.L., Masai H., Hartsuiker E., Dalgaard J.Z. (2015). The DNA-binding domain of *S. pombe* Mrc1 (claspin) acts to enhance stalling at replication barriers. PLoS ONE.

[74] Castel S.E., Ren J., Bhattacharjee S., Chang A.Y., Sanchez M., Valbuena A., Antequera F., Martienssen R.A. (2014). Dicer promotes transcription termination at sites of replication stress to maintain genome stability. Cell.

[75] Zaratiegui M., Castel S.E., Irvine D.V., Kloc A., Ren J., Li F., de Castro E., Marin L., Chang A.Y., Goto D. (2011). RNAi promotes heterochromatic silencing through replication-coupled release of RNA pol II. Nature.

[76] Pluta A.F., Mackay A.M., Ainsztein A.M., Goldberg I.G., Earnshaw W.C. (1995). The centromere: Hub of chromosomal activities. Science.

[77] Amor D.J., Kalitsis P., Sumer H., Choo K.H. (2004). Building the centromere: From foundation proteins to 3D organization. Trends Cell Biol..

[78] Morris C.A., Moazed D. (2007). Centromere assembly and propagation. Cell.

[79] Weaver B.A., Cleveland D.W. (2007). Aneuploidy: Instigator and inhibitor of tumorigenesis. Cancer Res..

[80] Greenfeder S.A., Newlon C.S. (1992). Replication forks pause at yeast centromeres. Mol. Cell. Biol..

[81] Smith J.G., Caddle M.S., Bulboaca G.H., Wohlgemuth J.G., Baum M., Clarke L., Calos M.P. (1995). Replication of centromere II of *Schizosaccharomyces pombe*. Mol. Cell. Biol..

[82] McIntosh J.R., Grishchuk E.L., West R.R. (2002). Chromosome-microtubule interactions during mitosis. Annu. Rev. Cell Dev. Biol..

[83] Bloom K., Yeh E. (2010). Tension management in the kinetochore. Curr. Biol..

[84] Fukagawa T., Earnshaw W.C. (2014). The centromere: Chromatin foundation for the kinetochore machinery. Dev. Cell.

[85] Ivessa A.S., Lenzmeier B.A., Bessler J.B., Goudsouzian L.K., Schnakenberg S.L., Zakian V.A. (2003). The *Saccharomyces cerevisiae* helicase Rrm3p facilitates replication past nonhistone protein-DNA complexes. Mol. Cell.

[86] Hodgson B., Calzada A., Labib K. (2007). Mrc1 and Tof1 regulate DNA replication forks in different ways during normal S phase. Mol. Biol. Cell.

[87] McAinsh A.D., Tytell J.D., Sorger P.K. (2003). Structure, function, and regulation of budding yeast kinetochores. Annu. Rev. Cell Dev. Biol..

[88] Mitra S., Gomez-Raja J., Larriba G., Dubey D.D., Sanyal K. (2014). Rad51-Rad52 mediated maintenance of centromeric chromatin in *candida albicans*. PLoS Genet..

[89] Carroll C.W., Straight A.F. (2006). Centromere formation: From epigenetics to self-assembly. Trends Cell Biol..

[90] Leman A.R., Noguchi E. (2013). The replication fork: Understanding the eukaryotic replication machinery and the challenges to genome duplication. Genes.

[91] Kim S.M., Dubey D.D., Huberman J.A. (2003). Early-replicating heterochromatin. Genes Dev..

[92] Hayashi M.T., Takahashi T.S., Nakagawa T., Nakayama J., Masukata H. (2009). The heterochromatin protein Swi6/Hp1 activates replication origins at the pericentromeric region and silent mating-type locus. Nat. Cell Biol..

[93] Li P.C., Chretien L., Cote J., Kelly T.J., Forsburg S.L. (2011). *S. pombe* replication protein Cdc18 (Cdc6) interacts with Swi6 (HP1) heterochromatin protein: Region specific effects and replication timing in the centromere. Cell Cycle.

[94] Shirayama M., Seth M., Lee H.C., Gu W., Ishidate T., Conte D., Mello C.C. (2012). piRNAs initiate an epigenetic memory of nonself RNA in the *C. elegans* germline. Cell.

[95] Law J.A., Jacobsen S.E. (2010). Establishing, maintaining and modifying DNA methylation patterns in plants and animals. Nat. Rev. Genet..

[96] Li P.C., Petreaca R.C., Jensen A., Yuan J.P., Green M.D., Forsburg S.L. (2013). Replication fork stability is essential for the maintenance of centromere integrity in the absence of heterochromatin. Cell Rep..

[97] Errico A., Aze A., Costanzo V. (2014). Mta2 promotes Tipin-dependent maintenance of replication fork integrity. Cell Cycle.

[98] Stirling P.C., Bloom M.S., Solanki-Patil T., Smith S., Sipahimalani P., Li Z., Kofoed M., Ben-Aroya S., Myung K., Hieter P. (2011). The complete spectrum of yeast chromosome instability genes identifies candidate CIN cancer genes and functional roles for ASTRA complex components. PLoS Genet..

[99] Li Z., Vizeacoumar F.J., Bahr S., Li J., Warringer J., Vizeacoumar F.S., Min R., Vandersluis B., Bellay J., Devit M. (2011). Systematic exploration of essential yeast gene function with temperature-sensitive mutants. Nat. Biotechnol..

[100] Cheng E., Vaisica J.A., Ou J., Baryshnikova A., Lu Y., Roth F.P., Brown G.W. (2012). Genome rearrangements caused by depletion of essential DNA replication proteins in *Saccharomyces cerevisiae*. Genetics.

[101] Lemoine F.J., Degtyareva N.P., Lobachev K., Petes T.D. (2005). Chromosomal translocations in yeast induced by low levels of DNA polymerase: A model for chromosome fragile sites. Cell.

[102] Lemoine F.J., Degtyareva N.P., Kokoska R.J., Petes T.D. (2008). Reduced levels of DNA polymerase δ induce chromosome fragile site instability in yeast. Mol. Cell. Biol..

[103] Admire A., Shanks L., Danzl N., Wang M., Weier U., Stevens W., Hunt E., Weinert T. (2006). Cycles of chromosome instability are associated with a fragile site and are increased by defects in DNA replication and checkpoint controls in yeast. Genes Dev..

[104] Roeder G.S., Fink G.R. (1980). DNA rearrangements associated with a transposable element in yeast. Cell.

[105] Argueso J.L., Westmoreland J., Mieczkowski P.A., Gawel M., Petes T.D., Resnick M.A. (2008). Double-strand breaks associated with repetitive DNA can reshape the genome. Proc. Natl. Acad. Sci. USA.

[106] Deshpande A.M., Newlon C.S. (1996). DNA replication fork pause sites dependent on transcription. Science.

[107] Soragni E., Kassavetis G.A. (2008). Absolute gene occupancies by RNA polymerase III, TFIIIB, and TFIIIC in *Saccharomyces cerevisiae*. J. Biol. Chem..

[108] Lowe T.M., Eddy S.R. (1997). Trnascan-se: A program for improved detection of transfer RNA genes in genomic sequence. Nucleic Acids Res..

[109] Lowe T.M. Genomic tRNA Database. http://lowelab.ucsc.edu/GtRNAdb/.

[110] McFarlane R.J., Whitehall S.K. (2009). tRNA genes in eukaryotic genome organization and reorganization. Cell Cycle.

[111] Haldar D., Kamakaka R.T. (2006). tRNA genes as chromatin barriers. Nat. Struct. Mol. Biol..

[112] Pryce D.W., Ramayah S., Jaendling A., McFarlane R.J. (2009). Recombination at DNA replication fork barriers is not universal and is differentially regulated by Swi1. Proc. Natl. Acad. Sci. USA.

[113] Usmanova MN T.N. (2008). Bioinformatic analysis of retroelement-associated sequences in human and mouse promoters. Proc. World Acad. Sci. Eng. Technol..

[114] Bochman M.L., Judge C.P., Zakian V.A. (2011). The Pif1 family in prokaryotes: What are our helicases doing in your bacteria?. Mol. Biol. Cell.

[115] de la Loza M.C., Wellinger R.E., Aguilera A. (2009). Stimulation of direct-repeat recombination by RNA polymerase III transcription. DNA Repair.

[116] Azvolinsky A., Dunaway S., Torres J.Z., Bessler J.B., Zakian V.A. (2006). The *S. cerevisiae* Rrm3p DNA helicase moves with the replication fork and affects replication of all yeast chromosomes. Genes Dev..

[117] Steinacher R., Osman F., Dalgaard J.Z., Lorenz A., Whitby M.C. (2012). The DNA helicase Pfh1 promotes fork merging at replication termination sites to ensure genome stability. Genes Dev..

[118] McDonald K.R., Guise A.J., Pourbozorgi-Langroudi P., Cristea I.M., Zakian V.A., Capra J.A., Sabouri N. (2016). Pfh1 is an accessory replicative helicase that interacts with the replisome to facilitate fork progression and preserve genome integrity. PLoS Genet..

[119] Thompson M., Haeusler R.A., Good P.D., Engelke D.R. (2003). Nucleolar clustering of dispersed tRNA genes. Science.

[120] Rozenzhak S., Mejia-Ramirez E., Williams J.S., Schaffer L., Hammond J.A., Head S.R., Russell P. (2010). Rad3 decorates critical chromosomal domains with γH2A to protect genome integrity during S-phase in fission yeast. PLoS Genet..

[121] Burns K.H., Boeke J.D. (2012). Human transposon tectonics. Cell.

[122] Bowen N.J., Jordan I.K., Epstein J.A., Wood V., Levin H.L. (2003). Retrotransposons and their recognition of pol II promoters: A comprehensive survey of the transposable elements from the complete genome sequence of *Schizosaccharomyces pombe*. Genome Res..

[123] Jacobs J.Z., Rosado-Lugo J.D., Cranz-Mileva S., Ciccaglione K.M., Tournier V., Zaratiegui M. (2015). Arrested replication forks guide retrotransposon integration. Science.

[124] Chatterjee A.G., Esnault C., Guo Y., Hung S., McQueen P.G., Levin H.L. (2014). Serial number tagging reveals a prominent sequence preference of retrotransposon integration. Nucleic Acids Res..

[125] Guo Y., Levin H.L. (2010). High-throughput sequencing of retrotransposon integration provides a saturated profile of target activity in *Schizosaccharomyces pombe*. Genome Res..

[126] Tsankov A., Yanagisawa Y., Rhind N., Regev A., Rando O.J. (2011). Evolutionary divergence of intrinsic and trans-regulated nucleosome positioning sequences reveals plastic rules for chromatin organization. Genome Res..

[127] Cam H.P., Noma K., Ebina H., Levin H.L., Grewal S.I. (2008). Host genome surveillance for retrotransposons by transposon-derived proteins. Nature.

[128] Feng G., Leem Y.E., Levin H.L. (2013). Transposon integration enhances expression of stress response genes. Nucleic Acids Res..

[129] Baller J.A., Gao J., Stamenova R., Curcio M.J., Voytas D.F. (2012). A nucleosomal surface defines an integration hotspot for the *Saccharomyces cerevisiae* Ty1 retrotransposon. Genome Res..

[130] Bridier-Nahmias A., Tchalikian-Cosson A., Baller J.A., Menouni R., Fayol H., Flores A., Saib A., Werner M., Voytas D.F., Lesage P. (2015). Retrotransposons. An RNA polymerase III subunit determines sites of retrotransposon integration. Science.

[131] Mularoni L., Zhou Y., Bowen T., Gangadharan S., Wheelan S.J., Boeke J.D. (2012). Retrotransposon Ty1 integration targets specifically positioned asymmetric nucleosomal DNA segments in tRNA hotspots. Genome Res..

[132] Dalgaard J.Z., Klar A.J. (2000). *swi1* and *swi3* perform imprinting, pausing, and termination of DNA replication in *S. pombe*. Cell.

[133] Eydmann T., Sommariva E., Inagawa T., Mian S., Klar A.J., Dalgaard J.Z. (2008). Rtf1-mediated eukaryotic site-specific replication termination. Genetics.

[134] Kaykov A., Holmes A.M., Arcangioli B. (2004). Formation, maintenance and consequences of the imprint at the mating-type locus in fission yeast. EMBO J..

[135] Vengrova S., Dalgaard J.Z. (2004). RNase-sensitive DNA modification(s) initiates *S. pombe* mating-type switching. Genes Dev..

[136] Holmes A., Roseaulin L., Schurra C., Waxin H., Lambert S., Zaratiegui M., Martienssen R.A., Arcangioli B. (2012). Lsd1 and Lsd2 control programmed replication fork pauses and imprinting in fission yeast. Cell Rep..

[137] Metzger E., Wissmann M., Yin N., Muller J.M., Schneider R., Peters A.H., Gunther T., Buettner R., Schule R. (2005). Lsd1 demethylates repressive histone marks to promote androgen-receptor-dependent transcription. Nature.

[138] Nicolas E., Lee M.G., Hakimi M.A., Cam H.P., Grewal S.I., Shiekhattar R. (2006). Fission yeast homologs of human histone H3 lysine 4 demethylase regulate a common set of genes with diverse functions. J. Biol. Chem..

[139] Shi Y., Lan F., Matson C., Mulligan P., Whetstine J.R., Cole P.A., Casero R.A., Shi Y. (2004). Histone demethylation mediated by the nuclear amine oxidase homolog Lsd1. Cell.

[140] Gordon M., Holt D.G., Panigrahi A., Wilhelm B.T., Erdjument-Bromage H., Tempst P., Bahler J., Cairns B.R. (2007). Genome-wide dynamics of SAPHIRE, an essential complex for gene activation and chromatin boundaries. Mol. Cell. Biol..

[141] Lan F., Zaratiegui M., Villen J., Vaughn M.W., Verdel A., Huarte M., Shi Y., Gygi S.P., Moazed D., Martienssen R.A. (2007). *S. pombe* Lsd1 homologs regulate heterochromatin propagation and euchromatic gene transcription. Mol. Cell.

[142] Cox R., Mirkin S.M. (1997). Characteristic enrichment of DNA repeats in different genomes. Proc. Natl. Acad. Sci. USA.

[143] Kremer E.J., Pritchard M., Lynch M., Yu S., Holman K., Baker E., Warren S.T., Schlessinger D., Sutherland G.R., Richards R.I. (1991). Mapping of DNA instability at the fragile X to a trinucleotide repeat sequence p(ccg)n. Science.

[144] The huntington’s disease collaborative research group (1993). A novel gene containing a trinucleotide repeat that is expanded and unstable on huntington’s disease chromosomes. Cell.

[145] Brook J.D., McCurrach M.E., Harley H.G., Buckler A.J., Church D., Aburatani H., Hunter K., Stanton V.P., Thirion J.P., Hudson T. (1992). Molecular basis of myotonic dystrophy: Expansion of a trinucleotide (CTG) repeat at the 3′ end of a transcript encoding a protein kinase family member. Cell.

[146] Liquori C.L., Ricker K., Moseley M.L., Jacobsen J.F., Kress W., Naylor S.L., Day J.W., Ranum L.P. (2001). Myotonic dystrophy type 2 caused by a CCTG expansion in intron 1 of ZNF9. Science.

[147] Matsuura T., Yamagata T., Burgess D.L., Rasmussen A., Grewal R.P., Watase K., Khajavi M., McCall A.E., Davis C.F., Zu L. (2000). Large expansion of the ATTCT pentanucleotide repeat in spinocerebellar ataxia type 10. Nat. Genet..

[148] Lalioti M.D., Scott H.S., Buresi C., Rossier C., Bottani A., Morris M.A., Malafosse A., Antonarakis S.E. (1997). Dodecamer repeat expansion in cystatin B gene in progressive myoclonus epilepsy. Nature.

[149] Frank-Kamenetskii M.D., Mirkin S.M. (1995). Triplex DNA structures. Annu. Rev. Biochem..

[150] Hile S.E., Eckert K.A. (2004). Positive correlation between DNA polymerase α-primase pausing and mutagenesis within polypyrimidine/polypurine microsatellite sequences. J. Mol. Biol..

[151] Hoyne P.R., Maher L.J. (2002). Functional studies of potential intrastrand triplex elements in the *Escherichia coli* genome. J. Mol. Biol..

[152] Krasilnikova M.M., Mirkin S.M. (2004). Replication stalling at friedreich’s ataxia (GAA)n repeats in vivo. Mol. Cell. Biol..

[153] Wang G., Carbajal S., Vijg J., DiGiovanni J., Vasquez K.M. (2008). DNA structure-induced genomic instability in vivo. J. Natl. Cancer Inst..

[154] Betous R., Rey L., Wang G., Pillaire M.J., Puget N., Selves J., Biard D.S., Shin-ya K., Vasquez K.M., Cazaux C. (2009). Role of TLS DNA polymerases eta and kappa in processing naturally occurring structured DNA in human cells. Mol. Carcinog..

[155] Diviacco S., Rapozzi V., Xodo L., Helene C., Quadrifoglio F., Giovannangeli C. (2001). Site-directed inhibition of DNA replication by triple helix formation. FASEB J..

[156] Liu G., Myers S., Chen X., Bissler J.J., Sinden R.R., Leffak M. (2012). Replication fork stalling and checkpoint activation by a PKD1 locus mirror repeat polypurine-polypyrimidine (Pu-Py) tract. J. Biol. Chem..

[157] Patel H.P., Lu L., Blaszak R.T., Bissler J.J. (2004). PKD1 intron 21: Triplex DNA formation and effect on replication. Nucleic Acids Res..

[158] Wang G., Vasquez K.M. (2009). Models for chromosomal replication-independent non-B DNA structure-induced genetic instability. Mol. Carcinog..

[159] Bochman M.L., Paeschke K., Zakian V.A. (2012). DNA secondary structures: Stability and function of G-quadruplex structures. Nat. Rev. Genet..

[160] Lopes J., Piazza A., Bermejo R., Kriegsman B., Colosio A., Teulade-Fichou M.P., Foiani M., Nicolas A. (2011). G-quadruplex-induced instability during leading-strand replication. EMBO J..

[161] Capra J.A., Paeschke K., Singh M., Zakian V.A. (2010). G-quadruplex DNA sequences are evolutionarily conserved and associated with distinct genomic features in *Saccharomyces cerevisiae*. PLoS Comput. Biol..

[162] Eddy J., Maizels N. (2006). Gene function correlates with potential for G4 DNA formation in the human genome. Nucleic Acids Res..

[163] Hershman S.G., Chen Q., Lee J.Y., Kozak M.L., Yue P., Wang L.S., Johnson F.B. (2008). Genomic distribution and functional analyses of potential G-quadruplex-forming sequences in *Saccharomyces cerevisiae*. Nucleic Acids Res..

[164] Kudlicki A.S. (2016). G-quadruplexes involving both strands of genomic DNA are highly abundant and colocalize with functional sites in the human genome. PLoS ONE.

[165] Kamath-Loeb A.S., Loeb L.A., Johansson E., Burgers P.M., Fry M. (2001). Interactions between the Werner syndrome helicase and DNA polymerase δ specifically facilitate copying of tetraplex and hairpin structures of the d(CGG)n trinucleotide repeat sequence. J. Biol. Chem..

[166] Woodford K.J., Howell R.M., Usdin K. (1994). A novel K(+)-dependent DNA synthesis arrest site in a commonly occurring sequence motif in eukaryotes. J. Biol. Chem..

[167] Kruisselbrink E., Guryev V., Brouwer K., Pontier D.B., Cuppen E., Tijsterman M. (2008). Mutagenic capacity of endogenous G4 DNA underlies genome instability in FANCJ-defective *C. elegans*. Curr. Biol..

[168] Williams J.D., Fleetwood S., Berroyer A., Kim N., Larson E.D. (2015). Sites of instability in the human TCF3 (E2A) gene adopt G-quadruplex DNA structures in vitro. Front. Genet..

[169] Edwards D.N., Machwe A., Wang Z., Orren D.K. (2014). Intramolecular telomeric G-quadruplexes dramatically inhibit DNA synthesis by replicative and translesion polymerases, revealing their potential to lead to genetic change. PLoS ONE.

[170] Trinh T.Q., Sinden R.R. (1991). Preferential DNA secondary structure mutagenesis in the lagging strand of replication in *E. coli*. Nature.

[171] Mendoza O., Bourdoncle A., Boule J.B., Brosh R.M., Mergny J.L. (2016). G-quadruplexes and helicases. Nucleic Acids Res..

[172] Duxin J.P., Walter J.C. (2015). What is the DNA repair defect underlying Fanconi anemia?. Curr. Opin. Cell Biol..

[173] Wu Y., Shin-ya K., Brosh R.M. (2008). FANCJ helicase defective in Fanconia anemia and breast cancer unwinds G-quadruplex DNA to defend genomic stability. Mol. Cell. Biol..

[174] Bharti S.K., Sommers J.A., George F., Kuper J., Hamon F., Shin-ya K., Teulade-Fichou M.P., Kisker C., Brosh R.M. (2013). Specialization among iron-sulfur cluster helicases to resolve G-quadruplex DNA structures that threaten genomic stability. J. Biol. Chem..

[175] Castillo Bosch P., Segura-Bayona S., Koole W., van Heteren J.T., Dewar J.M., Tijsterman M., Knipscheer P. (2014). FANCJ promotes DNA synthesis through G-quadruplex structures. EMBO J..

[176] Drosopoulos W.C., Kosiyatrakul S.T., Schildkraut C.L. (2015). BLM helicase facilitates telomere replication during leading strand synthesis of telomeres. J. Cell Biol..

[177] Suhasini A.N., Rawtani N.A., Wu Y., Sommers J.A., Sharma S., Mosedale G., North P.S., Cantor S.B., Hickson I.D., Brosh R.M. (2011). Interaction between the helicases genetically linked to Fanconi anemia group J and Bloom’s syndrome. EMBO J..

[178] Sarkies P., Murat P., Phillips L.G., Patel K.J., Balasubramanian S., Sale J.E. (2012). FANCJ coordinates two pathways that maintain epigenetic stability at G-quadruplex DNA. Nucleic Acids Res..

[179] Wallgren M., Mohammad J.B., Yan K.P., Pourbozorgi-Langroudi P., Ebrahimi M., Sabouri N. (2016). G-rich telomeric and ribosomal DNA sequences from the fission yeast genome form stable G-quadruplex DNA structures in vitro and are unwound by the Pfh1 DNA helicase. Nucleic Acids Res..

[180] Duan X.L., Liu N.N., Yang Y.T., Li H.H., Li M., Dou S.X., Xi X.G. (2015). G-quadruplexes significantly stimulate Pif1 helicase-catalyzed duplex DNA unwinding. J. Biol. Chem..

[181] Rhodes D., Lipps H.J. (2015). G-quadruplexes and their regulatory roles in biology. Nucleic Acids Res..

[182] Gray L.T., Vallur A.C., Eddy J., Maizels N. (2014). G quadruplexes are genomewide targets of transcriptional helicases XPB and XPD. Nat. Chem. Biol..

[183] Kendrick S., Hurley L.H. (2010). The role of G-quadruplex/i-motif secondary structures as *cis*-acting regulatory elements. Pure Appl. Chem..

[184] Besnard E., Babled A., Lapasset L., Milhavet O., Parrinello H., Dantec C., Marin J.M., Lemaitre J.M. (2012). Unraveling cell type-specific and reprogrammable human replication origin signatures associated with G-quadruplex consensus motifs. Nat. Struct. Mol. Biol..

[185] Comoglio F., Schlumpf T., Schmid V., Rohs R., Beisel C., Paro R. (2015). High-resolution profiling of Drosophila replication start sites reveals a DNA shape and chromatin signature of metazoan origins. Cell Rep..

[186] Valton A.L., Hassan-Zadeh V., Lema I., Boggetto N., Alberti P., Saintome C., Riou J.F., Prioleau M.N. (2014). G4 motifs affect origin positioning and efficiency in two vertebrate replicators. EMBO J..

[187] Gaillard H., Herrera-Moyano E., Aguilera A. (2013). Transcription-associated genome instability. Chem. Rev..

[188] Srivatsan A., Tehranchi A., MacAlpine D.M., Wang J.D. (2010). Co-orientation of replication and transcription preserves genome integrity. PLoS Genet..

[189] Vilette D., Ehrlich S.D., Michel B. (1996). Transcription-induced deletions in plasmid vectors: M13 DNA replication as a source of instability. Mol. Gen. Genet..

[190] Kotsantis P., Silva L.M., Irmscher S., Jones R.M., Folkes L., Gromak N., Petermann E. (2016). Increased global transcription activity as a mechanism of replication stress in cancer. Nat. Commun..

[191] Liu B., Alberts B.M. (1995). Head-on collision between a DNA replication apparatus and RNA polymerase transcription complex. Science.

[192] Mirkin E.V., Mirkin S.M. (2005). Mechanisms of transcription-replication collisions in bacteria. Mol. Cell. Biol..

[193] Prado F., Aguilera A. (2005). Impairment of replication fork progression mediates RNA polII transcription-associated recombination. EMBO J..

[194] Datta A., Jinks-Robertson S. (1995). Association of increased spontaneous mutation rates with high levels of transcription in yeast. Science.

[195] Kim N., Abdulovic A.L., Gealy R., Lippert M.J., Jinks-Robertson S. (2007). Transcription-associated mutagenesis in yeast is directly proportional to the level of gene expression and influenced by the direction of DNA replication. DNA Repair.

[196] French S. (1992). Consequences of replication fork movement through transcription units in vivo. Science.

[197] Wang J.D., Berkmen M.B., Grossman A.D. (2007). Genome-wide coorientation of replication and transcription reduces adverse effects on replication in *Bacillus subtilis*. Proc. Natl. Acad. Sci. USA.

[198] Postow L., Ullsperger C., Keller R.W., Bustamante C., Vologodskii A.V., Cozzarelli N.R. (2001). Positive torsional strain causes the formation of a four-way junction at replication forks. J. Biol. Chem..

[199] Guy L., Roten C.A. (2004). Genometric analyses of the organization of circular chromosomes: A universal pressure determines the direction of ribosomal RNA genes transcription relative to chromosome replication. Gene.

[200] Huvet M., Nicolay S., Touchon M., Audit B., d’Aubenton-Carafa Y., Arneodo A., Thermes C. (2007). Human gene organization driven by the coordination of replication and transcription. Genome Res..

[201] Wei X., Samarabandu J., Devdhar R.S., Siegel A.J., Acharya R., Berezney R. (1998). Segregation of transcription and replication sites into higher order domains. Science.

[202] Vieira K.F., Levings P.P., Hill M.A., Crusselle V.J., Kang S.H., Engel J.D., Bungert J. (2004). Recruitment of transcription complexes to the β-globin gene locus *in vivo* and *in vitro*. J. Biol. Chem..

[203] Smirnov E., Borkovec J., Kovacik L., Svidenska S., Schrofel A., Skalnikova M., Svindrych Z., Krizek P., Ovesny M., Hagen G.M. (2014). Separation of replication and transcription domains in nucleoli. J. Struct. Biol..

[204] Pliss A., Koberna K., Vecerova J., Malinsky J., Masata M., Fialova M., Raska I., Berezney R. (2005). Spatio-temporal dynamics at rDNA foci: Global switching between DNA replication and transcription. J. Cell. Biochem..

[205] Dimitrova D.S. (2011). DNA replication initiation patterns and spatial dynamics of the human ribosomal RNA gene loci. J. Cell Sci..

[206] Hernandez-Verdun D., Roussel P., Gebrane-Younes J. (2002). Emerging concepts of nucleolar assembly. J. Cell Sci..

[207] Busch H., Smetana K. (1970). The Nucleolus.

[208] Cabal G.G., Genovesio A., Rodriguez-Navarro S., Zimmer C., Gadal O., Lesne A., Buc H., Feuerbach-Fournier F., Olivo-Marin J.C., Hurt E.C. (2006). SAGA interacting factors confine sub-diffusion of transcribed genes to the nuclear envelope. Nature.

[209] Casolari J.M., Brown C.R., Komili S., West J., Hieronymus H., Silver P.A. (2004). Genome-wide localization of the nuclear transport machinery couples transcriptional status and nuclear organization. Cell.

[210] Elias-Arnanz M., Salas M. (1997). Bacteriophage ϕ29 DNA replication arrest caused by codirectional collisions with the transcription machinery. EMBO J..

[211] Azvolinsky A., Giresi P.G., Lieb J.D., Zakian V.A. (2009). Highly transcribed RNA polymerase II genes are impediments to replication fork progression in *Saccharomyces cerevisiae*. Mol. Cell.

[212] Huertas P., Garcia-Rubio M.L., Wellinger R.E., Luna R., Aguilera A. (2006). An *hpr1* point mutation that impairs transcription and mrnp biogenesis without increasing recombination. Mol. Cell. Biol..

[213] Aguilera A. (2002). The connection between transcription and genomic instability. EMBO J..

[214] Chavez S., Aguilera A. (1997). The yeast *hpr1* gene has a functional role in transcriptional elongation that uncovers a novel source of genome instability. Genes Dev..

[215] Mason P.B., Struhl K. (2005). Distinction and relationship between elongation rate and processivity of RNA polymerase II in vivo. Mol. Cell.

[216] Rondon A.G., Jimeno S., Garcia-Rubio M., Aguilera A. (2003). Molecular evidence that the eukaryotic THO/TREX complex is required for efficient transcription elongation. J. Biol. Chem..

[217] Strasser K., Masuda S., Mason P., Pfannstiel J., Oppizzi M., Rodriguez-Navarro S., Rondon A.G., Aguilera A., Struhl K., Reed R. (2002). TREX is a conserved complex coupling transcription with messenger RNA export. Nature.

[218] Zenklusen D., Vinciguerra P., Wyss J.C., Stutz F. (2002). Stable mRNP formation and export require cotranscriptional recruitment of the mRNA export factors Yra1p and Sub2p by Hpr1p. Mol. Cell. Biol..

[219] Labib K., Hodgson B. (2007). Replication fork barriers: Pausing for a break or stalling for time?. EMBO Rep..

[220] Omont N., Kepes F. (2004). Transcription/replication collisions cause bacterial transcription units to be longer on the leading strand of replication. Bioinformatics.

[221] Le Tallec B., Millot G.A., Blin M.E., Brison O., Dutrillaux B., Debatisse M. (2013). Common fragile site profiling in epithelial and erythroid cells reveals that most recurrent cancer deletions lie in fragile sites hosting large genes. Cell Rep..

[222] McAvoy S., Ganapathiraju S.C., Ducharme-Smith A.L., Pritchett J.R., Kosari F., Perez D.S., Zhu Y., James C.D., Smith D.I. (2007). Non-random inactivation of large common fragile site genes in different cancers. Cytogenet. Genome Res..

[223] Sabouri N., McDonald K.R., Webb C.J., Cristea I.M., Zakian V.A. (2012). DNA replication through hard-to-replicate sites, including both highly transcribed RNA pol II and pol III genes, requires the *S. pombe* Pfh1 helicase. Genes Dev..

[224] Tourriere H., Versini G., Cordon-Preciado V., Alabert C., Pasero P. (2005). Mrc1 and Tof1 promote replication fork progression and recovery independently of Rad53. Mol. Cell.

[225] Jinks-Robertson S., Bhagwat A.S. (2014). Transcription-associated mutagenesis. Annu. Rev. Genet..

[226] Pybus C., Pedraza-Reyes M., Ross C.A., Martin H., Ona K., Yasbin R.E., Robleto E. (2010). Transcription-associated mutation in *Bacillus subtilis* cells under stress. J. Bacteriol..

[227] Takahashi T., Burguiere-Slezak G., Van der Kemp P.A., Boiteux S. (2011). Topoisomerase 1 provokes the formation of short deletions in repeated sequences upon high transcription in *Saccharomyces cerevisiae*. Proc. Natl. Acad. Sci. USA.

[228] Hicks W.M., Kim M., Haber J.E. (2010). Increased mutagenesis and unique mutation signature associated with mitotic gene conversion. Science.

[229] Keil R.L., Roeder G.S. (1984). *Cis*-acting, recombination-stimulating activity in a fragment of the ribosomal DNA of *S. cerevisiae*. Cell.

[230] Grimm C., Schaer P., Munz P., Kohli J. (1991). The strong *adh1* promoter stimulates mitotic and meiotic recombination at the *ade6* gene of *Schizosaccharomyces pombe*. Mol. Cell. Biol..

[231] Thomas B.J., Rothstein R. (1989). Elevated recombination rates in transcriptionally active DNA. Cell.

[232] Huertas P., Aguilera A. (2003). Cotranscriptionally formed DNA:RNA hybrids mediate transcription elongation impairment and transcription-associated recombination. Mol. Cell.

[233] Garcia-Rubio M., Huertas P., Gonzalez-Barrera S., Aguilera A. (2003). Recombinogenic effects of DNA-damaging agents are synergistically increased by transcription in *Saccharomyces cerevisiae*. New insights into transcription-associated recombination. Genetics.

[234] Wang J.C. (1985). DNA topoisomerases. Annu. Rev. Biochem..

[235] Shen X., Mizuguchi G., Hamiche A., Wu C. (2000). A chromatin remodelling complex involved in transcription and DNA processing. Nature.

[236] Gan W., Guan Z., Liu J., Gui T., Shen K., Manley J.L., Li X. (2011). R-loop-mediated genomic instability is caused by impairment of replication fork progression. Genes Dev..

[237] Li X., Manley J.L. (2005). Inactivation of the SR protein splicing factor ASF/SF2 results in genomic instability. Cell.

[238] Santos-Pereira J.M., Garcia-Rubio M.L., Gonzalez-Aguilera C., Luna R., Aguilera A. (2014). A genome-wide function of THSC/TREX-2 at active genes prevents transcription-replication collisions. Nucleic Acids Res..

